# Analysis and Prediction of Pathways in HeLa Cells by Integrating Biological Levels of Organization with Systems-Biology Approaches

**DOI:** 10.1371/journal.pone.0065433

**Published:** 2013-06-10

**Authors:** Juan Carlos Higareda-Almaraz, Ilse A. Valtierra-Gutiérrez, Magdalena Hernandez-Ortiz, Sandra Contreras, Erika Hernandez, Sergio Encarnacion

**Affiliations:** 1 Functional Genomics of Prokaryotes Research Program, Center for Genomic Sciences, National Autonomous University of Mexico, Cuernavaca, Morelos, Mexico; 2 Undergraduate Program on Genomic Sciences, National Autonomous University of Mexico, Cuernavaca, Morelos, Mexico; University of Georgia, United States of America

## Abstract

It has recently begun to be considered that cancer is a systemic disease and that it must be studied at every level of complexity using many of the currently available approaches, including high-throughput technologies and bioinformatics. To achieve such understanding in cervical cancer, we collected information on gene, protein and phosphoprotein expression of the HeLa cell line and performed a comprehensive analysis of the different signaling pathways, transcription networks and metabolic events in which they participate. A total expression analysis by RNA-Seq of the HeLa cell line showed that 19,974 genes were transcribed. Of these, 3,360 were over-expressed, and 2,129 under-expressed when compared to the NHEK cell line. A protein-protein interaction network was derived from the over-expressed genes and used to identify central elements and, together with the analysis of over-represented transcription factor motifs, to predict active signaling and regulatory pathways. This was further validated by Metal-Oxide Affinity Chromatography (MOAC) and Tandem Mass Spectrometry (MS/MS) assays which retrieved phosphorylated proteins. The 14-3-3 family members emerge as important regulators in carcinogenesis and as possible clinical targets. We observed that the different over- and under-regulated pathways in cervical cancer could be interrelated through elements that participate in crosstalks, therefore belong to what we term “meta-pathways”. Additionally, we highlighted the relations of each one of the differentially represented pathways to one or more of the ten hallmarks of cancer. These features could be maintained in many other types of cancer, regardless of mutations or genomic rearrangements, and favor their robustness, adaptations and the evasion of tissue control. Probably, this could explain why cancer cells are not eliminated by selective pressure and why therapy trials directed against molecular targets are not as effective as expected.

## Introduction

Cells are complex, dynamic systems, which use molecular signaling circuits that govern basic cellular activities and coordinate their actions [1]. The ability of cells to perceive and respond in an appropriate manner to the microenvironment is the basis for homeostasis, development, tissue repair and immunity. Errors in information management are responsible for different cell-derived conditions, such as autoimmune diseases, metabolic syndromes and cancer [2]–[5].

Cancer requires a very complex set of conditions. It is driven by a Darwinian model of evolution at the cellular level [6], comprising all levels of cellular information (i.e., genetics, epigenetics, transcriptional and translational regulation and translational modifications). Accordingly, it involves communication between different cell types, and interactions between the tumoral microenvironment and the whole organism [7], [8]. Our understanding of cancer has evolved because of this context and acquired knowledge.

Hanahan and Weinberg suggested that all cancers have certain essential alterations in cell physiology that coordinate the malignant phenotype, which is characterized by self-sufficiency in growth signals, insensitivity to growth inhibitors, evasion of programmed cell death, increased replicative potential, sustained angiogenesis, tissue invasiveness and metastasis, reprogramming of energy metabolism and evasion of immune destruction. Moreover, these hallmarks are accompanied by additional enabling features, including mutations and genomic instability, and the promotion of inflammation by tumors [9], [10].

Cervical cancer represents an interesting opportunity for the study of malignant transformation, mainly due to our understanding of its etiologic agent, High-Risk Human Papilloma Viruses (HR-HPVs), which are found in 90.7% of cases [11]. The HR-HPV oncoproteins E6 and E7 are able to interact with the p53 and pRb tumor suppressors in addition to more than 300 other known proteins. Of the more than 120 types of HPVs that infect humans, only a few high-risk types are associated with carcinogenesis. HPV16 and HPV18 are the most prevalent high-risk HPVs, and they are present in 54.6% and 11% of cervical squamous cell carcinomas, respectively [12]–[14]. Patients with cancer caused by these HPV types are the most widely studied. The first established cervical carcinoma cell line, HeLa, is positive for HPV18 and has served as the basis for most of our knowledge regarding the underlying cell biology of cancer.

However, due to spontaneous elimination of the virus, not all patients infected with HR-HPV develop cervical cancer. Most HPV infections are subclinical, with only a small fraction producing epithelial lesions, and an even smaller fraction of these lesions developing into cancer [15]. Consequently, HR-HPV infection is necessary but not sufficient for the development of cervical cancer [16]. Thus, the conditions that allow the development of cervical cancer both following HR-HPV infection and in its absence are not thoroughly known.

Understanding biological complexity at different levels of organization (which would be critical for a model such as cancer, in which cellular dynamics are altered at the molecular and tissular levels) requires combining the results obtained from various experiments to recreate the system’s behavior [17], [18]. The molecular profiling methods known as “omics” (e.g., transcriptomics, proteomics, and metabolomics) allow a global search of the characteristics that define the system under study and the integration of this knowledge into simple models with great explanatory and predictive power. However, these models can and must be contrasted against new experimental data. Currently, the cellular signaling system represents the biggest challenge for systems biology [19]–[21].

A signaling pathway consists of multiple sequential events, including covalent modifications, recruitment, allosteric activation or inhibition and protein binding [22]. However, as our understanding of the interactions between signaling pathways increases, it becomes more apparent that the signals do not necessarily occur independently through parallel (and isolated) linear pathways, but rather, through a large and complex network of interconnected signaling pathways [23], [24].

The complex architecture of signaling networks can be understood as a set of interacting network motifs, which can provide specific network properties and add a new level of complexity to that which already exists within the spatio-temporal organization and compartmentalization of signals [25], [26].Network theory approaches have been useful to discriminate components that have a major overall effect on the system, given the number and variety of pathways in which they are involved [27]. Such highly-connected elements are relevant for both cellular homeostasis and disease [Hao et al. 2009]. A large number of efforts are being held in order to understand the key actors and pathways that facilitate the appearance and maintenance of cancer cells, and how these are physiologically related to the ten hallmarks proposed by Weinberg and Hanahan [10].

In a previous study, the analysis of the proteomes of six different cervical cancer cell lines and the protein-protein interaction networks in which they participated led us to propose that the delicate balance between the life and death decisions of cells, as well as the neoplastic phenotype, might be due to the overregulation of the transcription factors c-Myc and E2F1. This can apparently result from both viral infection and the overexpression of the protein 14-3-3Z, which has been shown to deregulate apoptosis and promote the G1 to S phase transition. Furthermore, it has been suggested to play an important role in the epithelial-mesenchymal transition (EMT) [28].Our study also gave insights into the multiple pathways that were orchestrated for the stabilization of the cancerous phenotype, but the actual relations among such pathways still needed to be analyzed from a broader perspective. In addition, the connections with transcriptional regulation were not fully exploited, and direct evidence of post-translational modifications that were transferred throughout signaling cascades was not yet provided.

The aim of the present study was to predict the behavior of signaling pathways and regulatory networks, and determine the molecular signature of cervical cancer in a HeLa cell line model. We used data generated via sequencing, performed a differential expression analysis and incorporated microarray data to predict the response of transcription factors. This information allowed the reconstruction of the signaling, metabolic and transcriptional regulation pathways. Finally, we enriched phosphorylated proteins using Metal-Oxide Affinity Chromatography (MOAC) and identified them using tandem mass spectrometry (MS/MS), in order to validate and build a model based on all these different levels of biological information.

## Results

**Figure 1:**
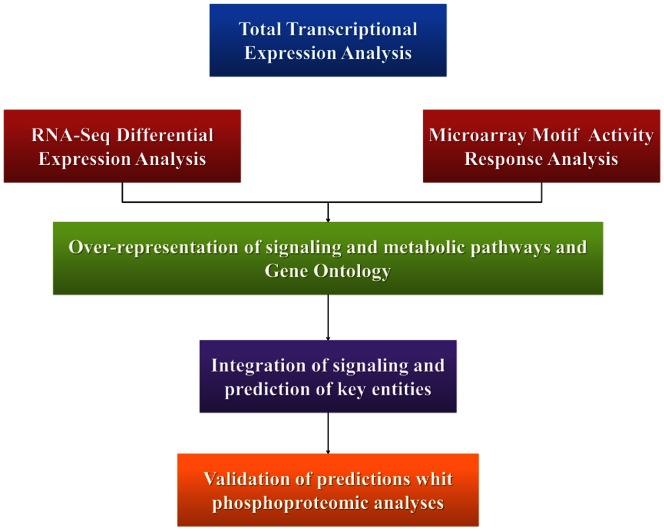
Pipeline. We have integrated different layers of information within biological cell dynamic that tracks the flow of information. First, we performed an analysis of all transcripts in HeLa cells; this analysis provided an overview of gene expression. Subsequently, we performed a RNA-seq differential expression analysis and a query of the over-representation of the activity of TFs. This allowed reconstruction of the metabolic pathways and the signaling and cellular transcriptional regulatory pathways. Finally, we validated this reconstruction with a phosphoproteomic analysis.

We integrated different layers of complexity within the dynamics of a HeLa cells in order to track the flow of information. Using this procedure, we were able to obtain information regarding the maintenance of the malignant state and the differences between cancerous and normal cells (Fig. 1).

### There are Different Gene Expression Profiles and Gene Ontologies in the HeLa and NHEK Cell Lines

**Figure 2:**
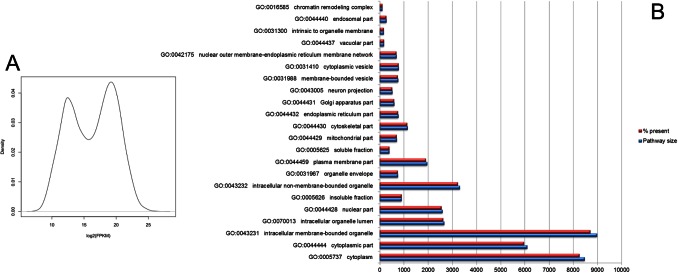
Gene expression patterns in the HeLa cells. a) The distribution of total transcripts shows that there are two populations, one low-abundance population and a second larger, high-abundance population. This dichotomy shows that the parameters used to search for low abundance transcripts was successful. b) A graphical representation of the cellular process that was distinguished based on Gene Ontology (GO), using the domain of cellular components in level 3; the amount of retrieved elements was compared against the total size of the pathway.

First, to assure the certainty of the gene expression profile of HeLa cells, we used the RNA-Seq data set generated by Nagaraj et al. [29]. This total expression analysis yielded a set of 19,974 transcripts. The principal difference between these analyses and those from the Nagaraj group was that we performed quartile normalization, which improves the accuracy of the differential expression calls for low-abundance transcripts by eliminating the bias of highly-expressed genes [30]. The distribution of the reads is bimodal (Fig. 2a). A gene was considered transcribed if the confidence interval lower boundary and the FPKM were greater than zero. In total 53934 genes were mapped from which 20110 are proteins. With the resulting full expression data, we developed a metric to elucidate the representation of cellular processes by means of Gene Ontology (GO) [31] using the domain of cellular components in level 3 (Fig. 2b). Importantly, the expression of each GO term fulfills at least 97% of the total reported for that term (Table S1 in File S1). We used this metric to establish the biological quality of our analysis and to build our predictions from these data, as well as to validate the differential expression analysis.

**Figure 3:**
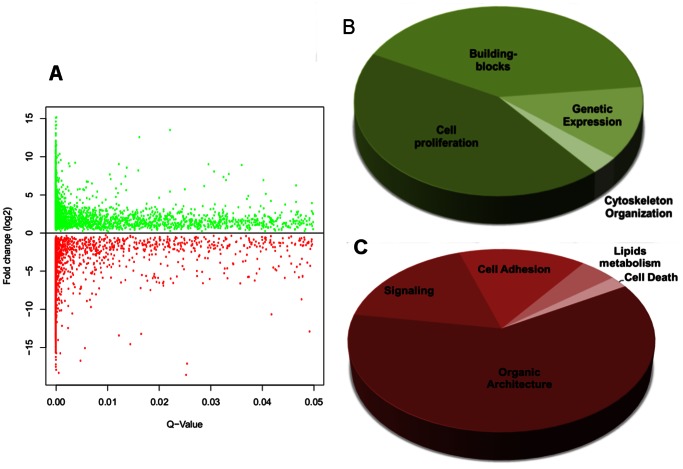
Differential gene expression in HeLa Cells versus NHEK. a) A scatter plot showing the quality of the RNA-seq differential expression analysis results, using the epithelial keratinocyte cell line NHEK as a control. There were a total of 3,360 overexpressed genes and 2,129 under-expressed genes. b) The percentage distribution of the level 3 biological process domain GO terms represented by the over-expressed transcripts. c) The percentage distribution of the level 3 biological process domain GO terms represented by the under-expressed transcripts. These charts were constructed from a summary of all the similar GO terms in a functional cellular circuit.

To understand the changes in gene expression in the HeLa cell line compared with a normal cell, we next performed a differential expression analysis via RNA-Seq, using the epithelial keratinocyte cell line NHEK as our expression control. Out of 47498 ENCODE-annotated transcripts in total, we identified 3,360 over-expressed genes and 2,129 under-expressed genes (Fig. 3a; Spreadsheet S1). Using this information, a GO enrichment analysis was built with the web tool ConsensusPathDB [32] using level 3 of the “Biological Process” domain. Even at this level of resolution, it was clear that the differential expression of genes in HeLa cells strongly favored cell proliferation over tissue organization (Table S2 in File S1). The categories with a clear over-representation were “Cell cycle”, “Gene expression”, “Metabolism building blocks” and “Cytoskeletal reorganization” (Fig. 3b). The categories that were under-represented included “Tissue development”, “Organs and systems”, “Signaling”, “Cell adhesion”, “Lipid metabolism” and “Programmed cell death” (Fig. 3c).

Based on these results, we propose that there is a strong tendency for HeLa cells to express genes that assist in the evasion of tissue control, which affords a clear adaptive advantage to proliferation without barriers.

### The Regulatory Network of Transcription Factors that are Differentially Expressed in HeLa Cells Controls Fundamental Processes Maintaining the Neoplastic State

To understand the transcriptional network that governs gene expression in HeLa cells, we used the Affymetrix microarray data from HeLa cells and normal cervical epithelia generated by the Scotto group [33] and deposited in the GEO database. These data were loaded into the MARA web tool [34], which retrieves the transcription factors with altered expression. 19,171 genes (corresponding to the Affymetrix HG-U133A annotation) were evaluated, and a total of 189 significantly-activated TFs were reported. The transcriptional targets of E2F, ZNF143, YY1, ELKA, GABP, NRF1, MYB, NFY, HIF1A, TFDP1 and ELF were over-represented, whereas the transcriptional targets of ETS, NFATc, NR1H4, SMAD, TFCP2, HIC1, AR, TBP, SRF and KLF12 were under-represented. Next, we used the database generated by the MARA transcriptional target analysis to establish the network of regulation obtained from the differential expression analysis. Targets were obtained for each TF, and their regulatory networks were reconstructed. It should be noted that c-Myc, hepatocyte nuclear factor 4-alpha, BRCA1, VHL and NEMO were all involved in more than one transcription factor network and were overexpressed.

**Figure 4:**
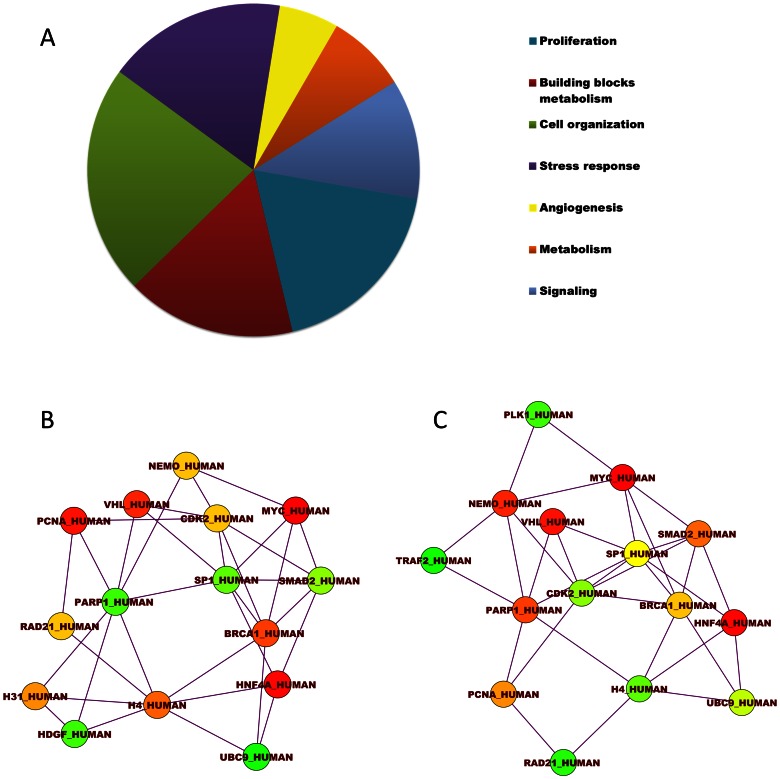
Transcription factor expression networks. a) The percentage distribution of level 3 biological process domain GO terms represented by the over-represented TF network, highlighting ”Cell proliferation”, ”Metabolism of building blocks”, ”Cellular organization”, ”Angiogenesis”, ”Central metabolism” and ”Signaling”. This chart was constructed from a summary of all the similar GO terms in a functional cellular circuit. b) Hubs that were obtained from the node degree centrality measure of the over-represented TF network. The color indicates the score, with red being the highest and yellow the lowest. c) Hubs were obtained from the betweeness centrality measure of the over-represented TF networks. The color indicates the score, with red being the highest and yellow the lowest.

After obtaining information from the TFs and their targets, we performed a GO enrichment analysis using the web tool ConsensusPathDB, and level 3 of the “Biological Process” domain. In the overexpressed-TF networks, we identified 103 GO terms (Table S3 in File S1), including “Cell proliferation”, “Metabolism of building blocks”, “Cellular organization”, “Angiogenesis”, “Central metabolism” and “Signaling” (Fig. 4a). In the under-expressed-TF networks, we identified 159 GO terms (Table S4 in File S1), which were largely related to “Tissue homeostasis” or “Miscellaneous”.

With the information generated by the TF network analysis, interaction networks were built using Cytoscape software [35] and the Hubba plug-in [36]. Hubs were defined using the node degree (Fig. 4b) and betweenness centralities (Fig. 4c). Surprisingly, c-Myc, HNF4A, BRCA1, VHL and NEMO were the nodes that had the highest values for these measures. These results suggest that a set of overexpressed genes in HeLa cells has control over a particular regulatory network that is not present in normal cervical epithelium. They also suggest reduced expression of some other regulatory networks. This feature may be an important source of the complexity that allows the strengthening of the cellular system by selection pressure.

### Identification of Under- or Over-represented Pathways that Govern the Neoplastic Phenotype in HeLa Cells

To understand which pathways and processes are responsible for maintaining the neoplastic phenotype, the complete list of previously identified over- and under-expressed transcripts was transformed into non-redundant UniProt identifiers [37]. A pathway enrichment analysis was then performed on this list using the ConsensusPathDB online tool. From the overexpressed transcripts, 83 over-represented pathways were recovered through the Pathway Interaction Database (PID) (Table S5 in File S1). Among these pathways, there were many remarkable signaling pathways, including those governed by ATR and Aurora A and B. Some transcriptional regulatory networks were identified as well, including “E2F”, “MYB”, “Targets of c-Myc transcriptional activation” and “Direct p53 effectors”. For the under-expressed transcripts, 15 over-represented pathways were detected via PID [38] (Table S6 in File S1). Some important pathways were the “Transcriptional targets of deltaNp63”, “TAp63”, “AP1 family members Fra1 and Fra2”, and “Direct p53 effectors”. Notably, both over- and under expressed transcripts were found within the “Direct p53 effectors” (Table S7 in File S1). Importantly, the identification of under-expressed and over-expressed transcripts from the same data set illustrates the power of our analysis, as it enabled us to distinguish, at the genomic level, components of the same network that promoted the neoplastic state either by actual participation or by omission.

Another pathway enrichment analysis was performed with a focus on the Kyoto Encyclopedia of Genes and Genomes (KEGG) [39] for both sets of transcripts; with this approach, we identified 17 pathways for the over-expressed genes (Table 1) and 25 pathways for the under-expressed genes (Table S8 in File S1). These results offer a perspective on the diverse events that occur in HeLa cells compared with non-malignant cells. Some of the highly represented processes were involved in cell proliferation (e.g., “DNA replication”, “Cell cycle” and “Homologous recombination”). Yet, other significant pathways included the “Fanconi Anemia pathway”, “Transcriptional misregulation in cancer” and “Small cell lung cancer”. These findings provided direct evidence of the conservation of malignant processes in different cancer types. Surprisingly, within the under-expressed genes, there was an over-representation of lipid metabolism pathways (particularly those involved in the metabolism of steroids, linoleic acid, arachidonic acid and the synthesis of unsaturated fatty acids), and pathways involved in cellular adhesion and several pathogen infections.

**Table 1:** Enriched PID pathway–based sets of over expressed transcripts.

Pathway name	Set size	Candidates	P-value	Q-value
DNA replication	36	28 (77.8%)	7.24E15	1.67E-12
Cell cycle	124	53 (42.7%)	9.42E11	1.09E-08
Homologous recombination	28	20 (71.4%)	7.71E10	5.94E-08
Systemic lupus erythematosus	138	51 (37.5%)	4.46E08	2.58E-06
Fanconi anemia pathway	52	26 (50.0%)	1.32E07	6.12E-06
Mismatch repair	23	15 (65.2%)	6.97E07	2.68E-05
Base excision repair	33	18 (54.5%)	2.27E06	7.49E-05
Transcriptional misregulation in cancer	180	53 (29.8%)	6.84E05	0.00198
Lysine degradation	49	20 (40.8%)	0.00018	0.0037
Purine metabolism	166	49 (29.5%)	0.00016	0.0037
One carbon pool by folate	19	10 (52.6%)	0.00062	0.0135
RNA transport	157	44 (28.0%)	0.00113	0.0217
HTLV-I infection	263	67 (25.5%)	0.00124	0.0221
Nucleotide excision repair	46	17 (37.0%)	0.00172	0.0284
Pyrimidine metabolism	101	30 (29.7%)	0.00253	0.039
Spliceosome	127	35 (27.6%)	0.00463	0.0668
Small cell lung cancer	87	25 (28.7%)	0.00876	0.119

The set size refers to the number of transcripts that have a UniProt ID in the corresponding PID pathway–based set at the ConsensusPathDB site. The number of candidates contained refers to the number of proteins that are part of the extended network and appear as part of the pathway. P-values were calculated using a hypergeometric test; Q-values represent a correction of the P-values for multiple testing using the false discovery rate method.

These data strengthened the results that were obtained from the MARA microarray analysis. However, even though the pathway enrichment analyses were important to our evaluation of the biological significance of the changes in gene expression, there are limits to these types of studies. For example, the same set of transcripts can be considered part of different pathways. Consequently, the results must be carefully evaluated and validated before any definitive interpretations are made. Therefore, similar to the current work, studies that involve different layers of information and validation are strongly needed.

### There is a Defined Pattern in the Activation of Cellular Circuits in HeLa Cells that Involves Interconnections and Crosstalk that could be Identified based on the Differentially Expressed Genes, Regulatory Networks and Pathways

To visualize the integration of the different layers of biological information that were obtained in the present work, the PID Batch Query tool was used. As an input, we used the list of transcripts obtained in the differential expression analysis, as well as the list of super-active TFs that was obtained from the MARA analysis. The PID batch query yielded 64 pathways curated in the Biopax level 3 format [40], which displayed an increased amount of interconnections and crosstalk among their cellular circuits. Such interconnection and crosstalk could allow constant proliferation, immortalization and cell migration by means of both the over- and under-expression of different genes and a pattern of transcriptional control that differed from that observed in normal cells.

**Figure 5:**
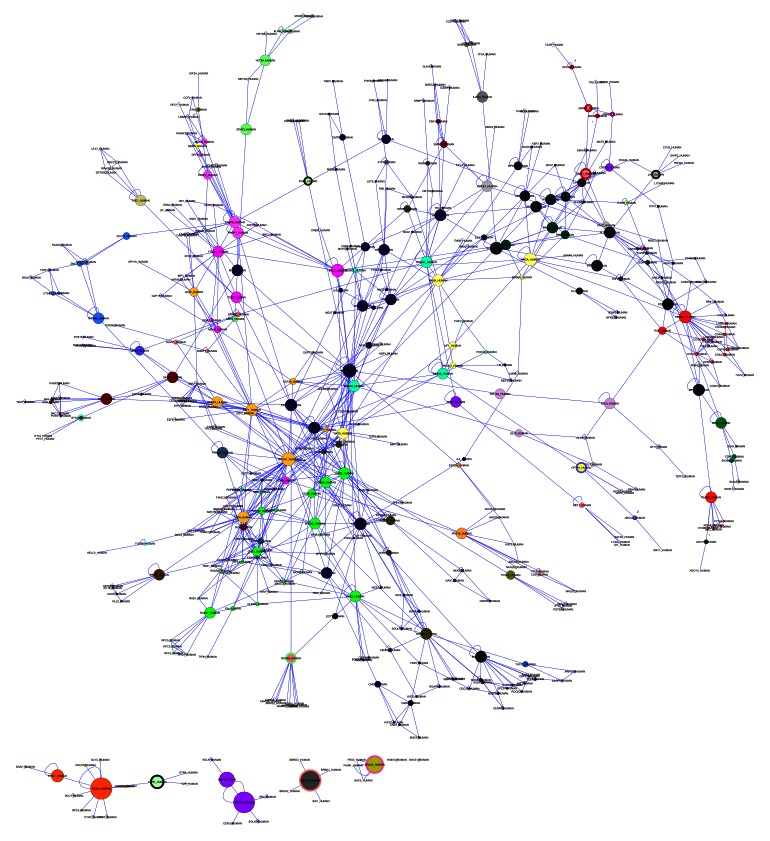
Network of interconnections and crosstalk among the cellular circuits. The network was made from data obtained by the analysis of signaling pathways and regulation. The nodes represent proteins that make up each of the pathways; each pathway is indicated by using different colors. The size of the node is determined by betweeness centrality measure, a larger size shows greater number of shortest paths that pass through that node.

To explore this hypothesis, we performed a reconstruction of every one of the aforementioned networks using the Bisogenet plug-in [41] in Cytoscape. Afterwards, we searched for the nodes that served as interconnectors between the explored pathways, and with the resulting data, we reconstructed two models. In the first model, we built a network by using every component of each pathway (Fig. 5). Node-degree and betweeness centralities were analyzed, and with these values, we could determine the presence of hubs in all the pathways. This lead to the observation that c-Myc, BRCA1, VEGFA and E2F1 were the most interconnected and influential nodes in all of the networks.

**Figure 6:**
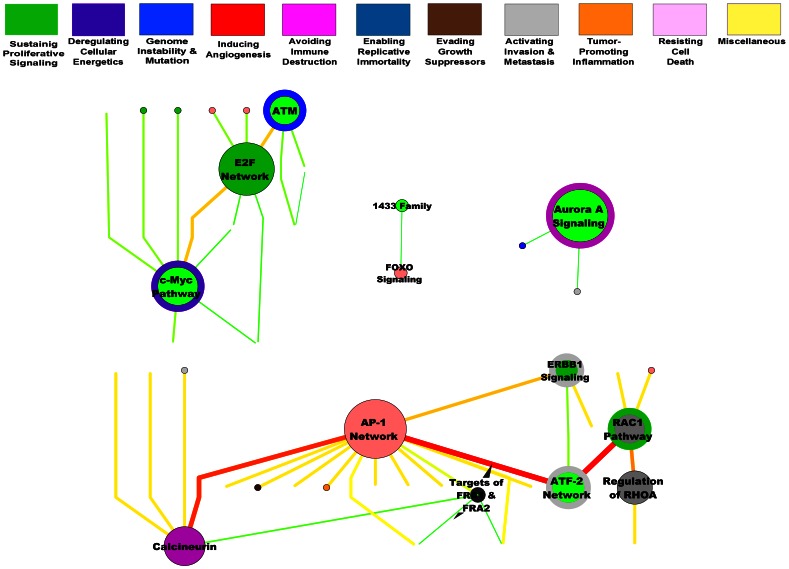
Meta-pathways analysis. An analysis was conducted combining the obtained signaling and transcriptional regulation pathways; the edges indicate the regulatory or hierarchical relationship, and the nodes indicate the pathway. The colors denote each of the hallmarks of cancer, with the two most representative hallmarks indicated per node. Additionally, we use a betwenness-weighed layout allowing the separation of dense clusters and the identification of elements with high centrality.

In our second model, we followed the hypothesis that there are well-defined cellular circuits that can be extrapolated to other malignant cells. Such circuits can be adapted to the concept of “Hallmarks of Cancer” that was originally proposed by Hanahan and Weinberg, which we reshaped in what we called “meta-pathways” (Fig. 6, Figure S1). Each one of the pathways that appear in the figure possesses a certain degree of connection with other pathways that was determined either by transcriptional regulation, signaling, metabolism or a combination of these mechanisms. This meta-network would be capable of maintaining the neoplastic phenotype. In Figure 6, each of the pathways and meta-pathways is related to its possible role in the generation of the hallmarks of cancer.

To validate the data of pathway over- and under- representation that were obtained, we conducted a phosphoproteomic analysis of the HeLa cell line via enrichment with Metal-Oxide Affinity Chromatography followed by identification of the proteins via LC/MS-MS. We identified a total of 271 phosphorylated proteins (Table S9 in File S1), reported in 40 level 3 GO terms (Table S10 in File S1), 21 PID pathways (Table S11 in File S1) and 16 KEGG pathways (Table S12 in File S1). As expected, due to the well-documented low correlation between transcript expression and protein expression, only 17% of the proteins had their equivalence as a transcript. In contrast, we found that there was a high correlation between the pathways which were based on transcript/TF expression and those based on phosphorylated proteins. Significantly, among these groups were “Validated targets of c-Myc transcriptional activation”, “Signaling events mediated by HDAC Class III”, “LKB1 signaling events”, “Class I P3K signaling events” and “FoxO family signaling”. However, we can find some important pathways that encompass members of the three levels of biological information. For example, the “c-Myb transcription factor network” and the “E2F transcription factor network”, whose targets were found within “c-Myc transcriptional activation” and, simultaneously, in pathways of central metabolism.

**Figure 7:**
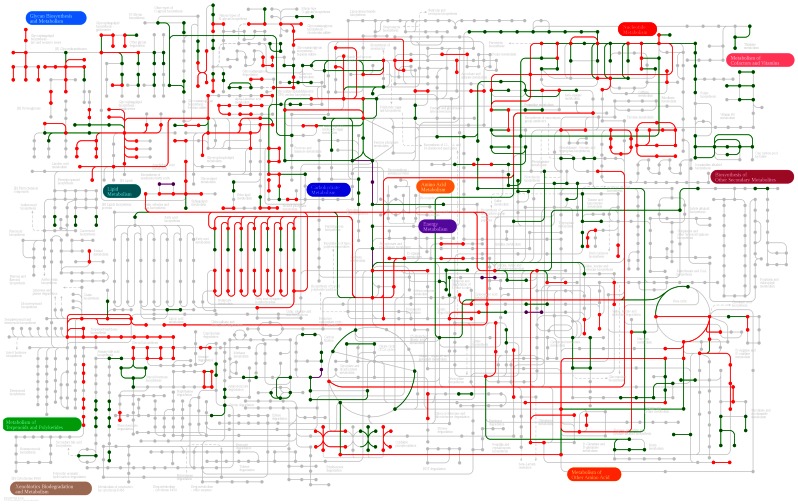
Over-represented metabolic pathways. Metabolic maps were reconstructed based on the KEGG database and the over-represented TF networks and identified phosphoprotein analyses, resulting in a map of general metabolism. The over-expressed transcripts are displayed in green, the under-expressed transcripts in red and the identified phosphoproteins in purple.

**Figure 8:**
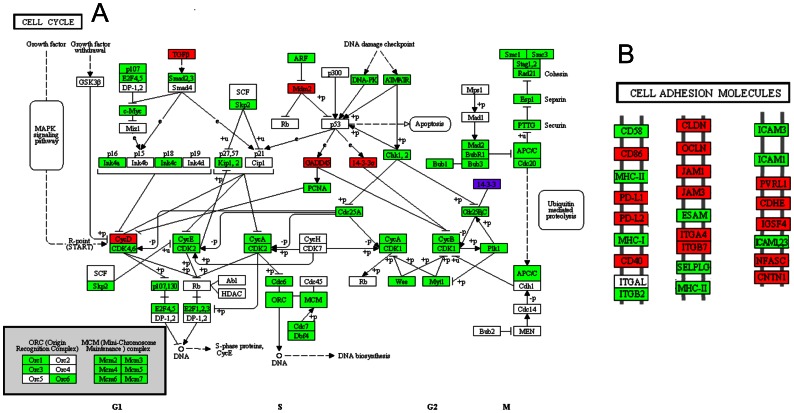
Cell cycle and cell adhesion molecules pathways. a) A reconstruction of cell cycle and MCM complex pathways. b) A reconstruction of cell adhesion molecules. Both maps were constructed based on the KEGG database and the transcriptomics, over-represented TF networks and identified-phosphoprotein analyses. The overexpressed transcripts are displayed in green, the under-expressed transcripts in red and the identified phosphoproteins in purple.

**Figure 9:**
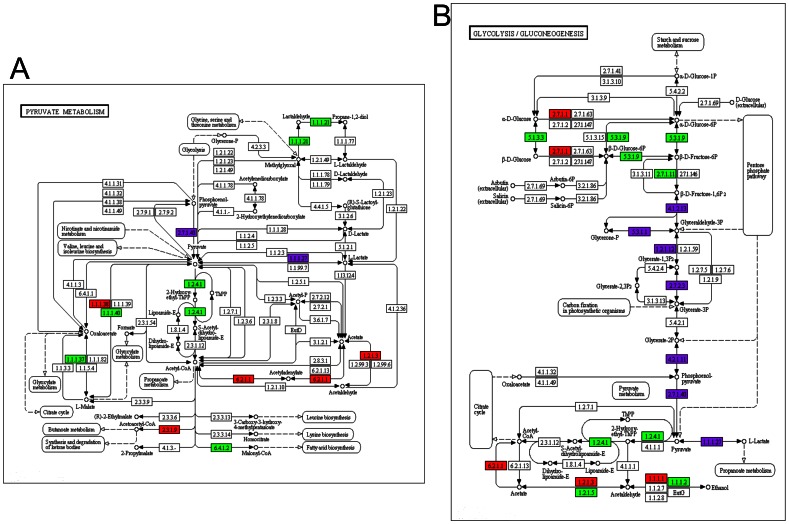
Over-represented metabolic pathways. Metabolic maps were reconstructed based on the KEGG database and the over-represented TF networks and identified phosphoprotein analyses, resulting in a map of a) pyruvate metabolism and b) glycolysis. The over-expressed transcripts are displayed in green, the under-expressed transcripts in red and the identified phosphoproteins in purple.

Finally, the entire data set that was extracted from the three layers of information was used to reconstruct the signaling, regulatory and metabolic networks that govern the HeLa cell line and are different from the networks present in both normal cervical epithelium and the NHEK keratinocyte cell line. For this purpose, the KEGG Mapper tool [42] was used to assemble the general metabolic map (Fig. 7), the cell cycle map (Fig. 8a) and the adhesion molecules and focal adhesion maps (Fig. 8b). Based on these maps, we were able to identify a clear pattern that could be divided into at least three groups with the following characteristics:

1. An increase in cell proliferation, resulting from the overexpression of the MCM-complex genes and diverse cell-cycle–associated proteins, which permitted sustained proliferative signaling.
2. Over-activation of carbohydrate metabolism (e.g., glycolysis and gluconeogenesis) (Fig. 9b), as well as an increase in the expression of enzymes in the pentose-phosphate pathway, which generates carbon skeletons for the synthesis of nitrogenous bases and histidine. In addition, malonyl-CoA can be obtained from pyruvate metabolism (Fig. 9a), which matches the “deregulation of cellular energetics” hallmark of cancer.
3. Activation of invasion and metastasis that was caused by the loss/gain of expression of several cell adhesion proteins, such as CLDN, OCLN and ESAM, the expression of which was increased.

## Discussion

In the present work, we performed an analysis of the different layers of biological information available from HeLa cells. The purpose of this analysis was to build a biological model of the pathways and meta-pathways that would allow us to define the relationships between this model and the hallmarks of cancer while maintaining a systemic perspective.

Hanahan and Weinberg postulated the existence of an intricate cellular circuitry that can be individually aligned with the hallmarks of cancer. They also proposed that in the near future, every circuit might be segmented into specialized sub-circuits that support discrete biological properties in normal cells and are reprogrammed in cancer cells, resulting in their hallmark capabilities. The work presented here is an attempt to prove, both theoretically and experimentally, the existence of these sub-circuits and their relationships to the specific pathways that afford specific cellular functions. Previous studies have attempted to integrate different levels of information for the prediction of signaling pathways that are either a determinant of or an obstacle to the malignant state.

Minn et al. [43] designed a novel methodology that permits the definition of new signaling pathways. This methodology is based on the initial identification of a gene that works as a master regulator. Next, the available information is increased via a gene set analysis (GSEA). Finally, the regulatory interactions are functionally validated via the over-expression of some genes that participate in the proposed pathway. This strategy can be coupled to a systemic analysis, such as our own, to validate novel or non-canonical pathways.

The validation step is critical because it has to be thoroughly representative of the complete pathways network, and the most frequently used technique for validation is the direct measurement of mRNA or proteins [44]. Therefore, we decided to validate our initial predictions using proteomics, with a special emphasis on identifying phosphoproteins. The phosphoproteomic validation was pursued because reversible phosphorylation of proteins is the most widely known, and presumably the most frequently used, post-translational modification in mammalian cells. Approximately 1.7% of the human genome encodes protein kinases [45], and at any moment, approximately 30% of all proteins can be phosphorylated [46]. Phosphorylation suggests that the protein is in its active form (as is the case with several metabolic enzymes) and that upstream signals are active. Alternatively, phosphorylation could result in a conformational change that allows the modulation of specific activities [47].

The analysis of total transcripts in the HeLa cell line yielded a notable result. A total of 19,974 genes were found to be transcribed, and while this may appear to be an exaggerated number, it has been demonstrated via RNA-Seq analysis that approximately 16,245 genes are transcribed in several types of breast cancer, and this number can vary from 14,648 to 18,290 [48]. When we conducted the GO analysis, we observed that the transcripts covered at least 97% of the GO terms. This aided in the evaluation of the quality of the information we obtained and prepared us to make the comparisons with the differential expression data.

When analyzing the differential-expression data from a classical perspective, we observed over-expression of the typical oncogenic proteins that participate in diverse types of cancer. These proteins make up three groups: 1) the c-Myc and c-Myb transcription factors, which are expressed in a great variety of tumors [49]–[52], 2) the DNA repair and recombination proteins BRCA1 and 2, which are amplified in breast cancer and non-small-cell lung cancer [53], [54], and 3) the proteins assigned to mitotic checkpoints, such as BUB1 and BUB3, which can result in genomic instability when overexpressed [55], [56].

Within the global perspective of this work, we found 3,360 overexpressed genes and 2,129 under expressed genes. The GOs and pathways that resulted from the ConsensusPathDB analysis showed a clear over-expression of genes that are coadjuvant in maintaining the sustained proliferative signaling cancer hallmark at the transcriptional, signaling or metabolic levels.

Similarly, when we analyzed the data regarding all the hyperactive transcription factors, we clearly found that the E2F, c-Myc and c-Myb pathways were positively regulating a large number of genes that initiate mitosis and allow cell cycle progression [57]–[59]. The over-expression of the FOXM1 transcription factor network has been associated with increased cell proliferation in animal models of prostate carcinoma [60]. Through the integration and validation of these data via the phosphoproteomic analysis, we found that over 70% of the components of the “Cell Cycle” KEGG pathway (Fig. 7a) and all of the components of the Minichromosome Maintenance Complex, whose increased levels have been observed in two other cancer models [61], [62], were over-expressed.

Regarding the deregulation of cellular energetics, we observed a clear increase in glycolysis and pyruvate/lactate metabolism, which is expelled from the cell, resulting in the acidification and remodeling of the extracellular matrix [63], [64]. However, the energetic inefficiency of using glycolysis to obtain ATP is compensated by the synthesis of substrates to create cellular building blocks (i.e., lipids, nitrogenous bases and peptides) from carbon skeletons that are obtained from glucose [65], [66] (Fig. 6).

One of the key enzymes in cancer is Pyruvate Kinase M2 because it possesses an extra tyrosine that is phosphorylated, which allows its detection in the phosphoproteomic analysis. This phosphorylation inhibits the positive regulation resulting from the fructose-1,6-bisphosphate level, stimulating the pathway and leaving a large amount of phosphorylated intermediates that can be used for anabolic synthesis and cell growth [67]. On the other hand, the huge quantity of glucose that is required by the cell to obtain energy is facilitated by the over-expression of the genes that encode the membrane glucose transporters GTR3, GTR4, GTR8 and GTR14 [68]. Glucose remains inside of the cell because it is phosphorylated and converted into glucose-6-phosphate by HXK2, which is also over-expressed in HeLa cells. During this step, glucose-6-phosphate is shunted into the pentose-phosphate pathway and is used for nitrogenous base metabolism, and we found that the enzymes responsible for turning glucose-6-phosphate into riboses and deoxyriboses [69] (i.e., G6PI, K6PF, DEOC, RBSK, KPRA, KPRB and PRPS1) were all over-expressed. At the transcriptional level, most of metabolic enzymes are regulated by the action of the c-Myc and HIF-1-alpha transcriptional regulatory networks [70], which were over-expressed at the transcriptional, TF and phosphoprotein levels.

The “Activation, Invasion and Metastasis” hallmark of cancer is profoundly complex because it reflects the differential expression of diverse adhesion molecules, such as the Claudins. These molecules are involved in a highly complex interplay and have been reported to be both over- and under-expressed in other malignancies (Fig. 7b) [71]. One indication of metastatic potential is the appearance of EMT markers. In the present study, we detected the over-expression of OCLN, VIME and 14-3-3Z [72], [73]. As for transcriptional regulation, the Endotelins pathway was over-regulated due to the expression of EDN2 and EDNRA, which resulted in the crossroads between migration and cell proliferation. Finally, the “Signaling events mediated by VEGF” pathway was over-activated because of the over-expression of VEGFA and VEGFB. This pathway permits angiogenesis, vasculogenesis and endothelial cell growth. Furthermore, it represents a crosstalk among various cancer hallmarks because it induces endothelial cell proliferation, promotes cell migration, inhibits apoptosis and induces permeabilization of blood vessels [74].

At the protein level, the family of 14-3-3 signal transducers plays an important role, given that their differential expression affects cell proliferation, evasion of apoptosis, cell adhesion, mitogenic signaling and the EMT [75]–[78]. The 14-3-3S isoform was found to be under-expressed in the differential expression analysis. This decreased expression supports most models that suggest that this protein functions as a tumor suppressor, and its loss or reduced expression is strongly correlated with a poor prognosis [79]. It is induced by DNA damage and is required for stable G2 arrest. It is directly regulated by p53 and has been found to be silenced or diminished in the majority proportion of carcinomas. The inactivation of 14-3-3S also leads to the immortalization of primary keratinocytes [80].

At the phosphoprotein level we identified the 14-3-3B, 14-3-3E, 14-3-3F, 14-3-3G, 14-3-3S and 14-3-3Z isoforms. The 14-3-3Z isoform antagonizes the 14-3-3S isoform; the amount of the latter diminishes because of the decrease in the expression of p53 in breast cancer, and its overexpression has been correlated with the EMT, metastasis and cell proliferation. Thus, it has been proposed that this family of signal-transducing proteins is critical for the malignant phenotype [81], [82].

**Figure 10:**
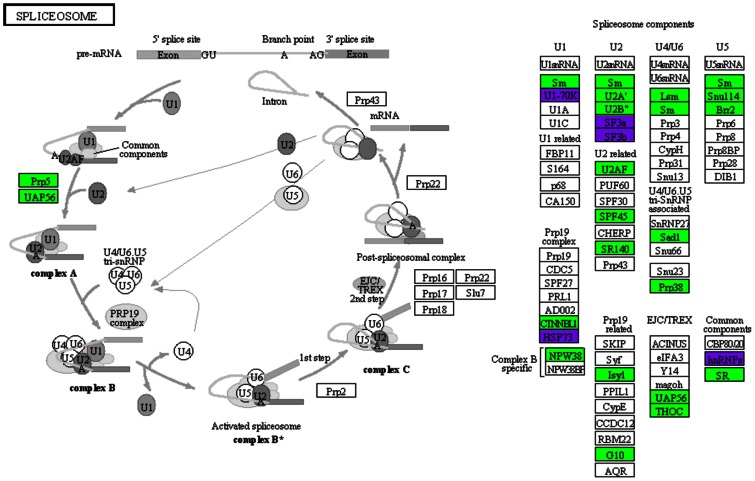
Spliceosome pathway. The spliceosome pathway was reconstructed based on the KEGG database and the transcriptomics, over-represented TF networks and identified phosphoprotein analyses. The over-expressed transcripts are displayed in green, the under-expressed transcripts in red and the identified phosphoproteins in purple.

After conducting the integrative analysis, we discovered that a large number of spliceosome genes were over-expressed. Constituents of the U2 component, including SRSF1, which is considered oncogenic itself, were particularly over-expressed. It has been suggested that any change in the stoichiometry or activity of splicing factors is capable of modifying the proportions of isoforms that normally do not exist or are less abundant in normal cells (Fig. 10). This phenomenon could contribute directly or indirectly to the development, progression and maintenance of cancer. Another hypothesis proposes that diverse RNA-binding proteins possess a wide array of functions, and changes in their expression could trigger oncogenic effects that are unrelated to their original role within the spliceosome [18], [83].

Integrating the evidence we obtained at the systems level, we have created a model to show the relationship between the hallmarks of cancer and the signaling, regulatory and metabolic pathways that are differentially expressed in HeLa cells. Figure 6 and Supplementary Figure 1 illustrate what we have termed “meta-pathways.” In these meta-pathways, the reconstructed pathways show their interrelations at the gene regulation, signaling and/or metabolic levels. Each node represents a pathway and is colored according to the two most representative hallmarks. One of the first features that stood out is the large number of signals that sustain the proliferative state of HeLa cells, as well as the great redundancy that can be appreciated among the different hallmarks. These data suggest that this system is extremely robust, as every hallmark is represented more than once and is supported by different pathways. Such behavior would provide an explanation for why directed therapies have not had the expected level of success. When one gene or subset of genes collapses, a bottleneck would immediately develop. This might result in the positive selection of a phenotype that could maintain the hallmarks of cancer via other pathways [84], [85].

Using the data generated in the present work, we propose that systematic robustness is not entirely random. Enough evidence was found to suggest that there are defined patterns in the activation of cellular circuits that involve interconnections and interferences associated to the hallmarks of cancer, the latter being accurately represented by the meta-pathways we have proposed. These observations allow us to hypothesize that, when a cell expresses abnormal levels of key proteins (e.g., TFs, signal transducers, metabolic enzymes, splicing factors or whole pathways) that should normally be repressed, it is highly likely that, if any of these expression patterns are advantageous for the cell, such patterns will become fixed by selection pressure. These effects will allow the cell to possess a response arsenal that can become more sophisticated with further exposure to selective pressures over time.

## Methods

### Gene and Transcript Quantification

Raw reads were obtained from the European Nucleotide Archive (ENA) under the study accession ERP000959. This data was described in Nagaraj et al, 2011. This data was sequenced on two Illumina Genome Analyzer IIx lanes using 76+7 cycles.

Raw reads of two sequencing lanes were combined, adapters were trimmed, and reads shorter than 70 nt, or with more than five bases below a quality score of 15 (PHRED-scale) were removed. The processed reads were aligned to the human reference genome (hg19/GRCh37 excluding additional haplotypes) using TopHat v1.0.13 [86] and transcripts and genes of the Ensembl [87] release 59 were quantified using Cufflinks v0.8.3 [88].

50 million single-end 76 bp reads were mapped to the human reference sequence and assembled into 183086 Transcripts [89].

### Differential Expression Analysis from RNA-seq Data

Paired-end RNA-seq data of HeLa-S3 and NHEK cell-lines was downloaded from the ENCyclopediaOf DNA Elements (ENCODE) [90] project’s webpage (UCSC accession numbers wgEncodeEH000130 and wgEncodeEH000131 respectively). Fastq files from two HeLa-S3 75×75 paired-end RNA-seq libraries (experiment numbers 10881, 10882) and two NHEK libraries (experiment numbers 10884, 11586) were aligned to the hg19 version of the human reference genome using TopHat v1.4.1 and Gencode annotation Version 12. Default parameters were used and only the read length was modified to 75 bases. The human genome index was built using bowtie v0.12.7 [89]. Differentially expressed genes between NHEK and HeLa-S3 were identified using cuffdiff v 1.3.0 from the Cufflinks package. A P-value threshold of 0.01 was set for all significant differentially-expressed genes.

### Transcriptional Factor Analysis

Affymetrix microarray dataset HG-U133A of HeLa and normal epithelium was downloaded from the Gene Expression Omnibus (GEO) [91] with accession numbers GSM246123 and GSM246422 respectively. The.cel files were uploaded to the website of MARA. This algorithm normalizes them altogether, assigns PolII promoters and binding sites within them to probe sets present on the microarray, and runs the TF activity analysis.

### Association of Transcriptional Targets with their RNA Expression Level

The information of the genes that were reported as targets of each differentially activated TF, as reported by MARA, was associated with their particular levels of expression according to the RNA-Seq analysis. Individual MARA reports for each gene were parsed through ad-hoc scripts written in Perl 5.12.4. The processed information, as well as the data from the RNA-Seq results, were poured into a relational database built in MySQL Server 5.5 (Community Edition). Subsequently, a query for each relevant TF was conducted, relating the targets that corresponded to every TF with RNA-Seq data by joining the corresponding UniProt identifiers. The outputs were stored in plain text files and analyzed with spreadsheet software.

### Network Reconstruction

The network reconstruction was performed with the aid of the Cytoscape plug-in BisoGenet, using the identified proteins as bait nodes and adding edges with the following parameters: Organism> Homo sapiens, protein identifiers only; Data Settings>protein-protein interactions; all data sources and all experimental methods; method> By adding edges connecting input nodes and as Output>Proteins.

### Pathway and GO Enrichment Analysis

Enrichment was done employing ConsensusPathDB, of the Max Planck Institute for Molecular Genetics, by using the overrepresentation analysis online tool. As input, we uploaded the UNIPROT protein identifiers of all the elements of the total expression analysis, differential expression analysis, TF networks and identified phosphoproteins. We searched for pathways as defined by PID and KEGG, with a minimal overlap with the input list of 5 and a P-value cutoff at 0.0001. Also, employing the same website and the same analysis tool, we performed an enrichment analysis based on Gene Ontology level 3 categories of “Biological processes”. For this analysis, we considered only the identified core proteins and set the p-value cutoff at 0.00001.

### Cell Culture

The HeLa cell line was provided by the oncology laboratory of the Centro Médico Siglo XXI which belongs to the Instituto Mexicano del Seguro Social. The HeLa cell line was cultured in RPMI-advanced 1640 serum-free media (Gibco BRL, USA) with red phenol and antibiotic-antimycotic solution (10,000 units penicillin, 10 mg streptomycin, and 25 µg amphotericin B per mL), supplemented with 1% fetal bovine serum (Invitrogen, Carlsbad, CA) and 200 mM of GlutaMAX (Invitrogen). The cells were incubated in 5% of CO_2_ and humidity saturation at 37°C in culture flasks of 75 cm^2^ (NalgeNunc International, Rochester, NY). Cells were harvested at 70% confluence with Verseno solution (Tris base 25 mM, NaCL 136.8 mM, KCl 5.36 mM, EDTA 1 mM pH7.7) and washed 3 times in phosphate buffer saline (0.1 M sodium phosphate and 0.15 M NaCl in one liter, pH 7.2).

### Enrichment of Phosphoproteins

Protein phenol extraction was performed [92]. The protein extraction was resuspended in incubation buffer and then the metal-oxide affinity chromatography (MOAC) [93]. The phospho-protein fraction was eluted and precipitated [94], and then a Sodium dodecyl sulfate-polyacrylamide Gel Electrophoresis (SDS-PAGE) was conducted. Gels were stained with Coomassie Blue G-250. All bands were excised with a razor blade and the tryptic digestion was performed.

### Chromatographic Separation

The tryptic peptides (8 ul) were desalted and concentrated on a Zorbax (Agilent 5065-9913) prior to analysis on a reverse-phase column (Agilent Zorbax 300SB C18, 3.5 um, 150×0.075 mm). Separation was performed at 400 nL/min using a lineal gradient. Mobile phase A was water with 0.1% formic acid by volume. Mobile phase B was acetonitrile with 0.1% formic acid by volume. The gradient conditions in the chromatographic run were set up as follow: A 95% (0 min) to 95% (14 min); A 95%(14 min) to 60% (54 min); A 60% (54 min) to 20% (56 min); A 20% (56 min) to 20% (61 min); A 20% (61 min) to 95% (62 min); and A 95% (62 min) to 95% (72 min).

### MS/MS Analysis

Proteins were analyzed by MS/MS using a nanoflow chromatograph (Agilent 1100 nano pump G2226A) coupled to a hybrid triple quadrupole linear ion trap (QTRAP 3200, AB Sciex) equipped with a Nanospray II source and using Information Dependent Acquisition (IDA). Precursor ion determination was carried out using an Enhanced MS scan over a mass range of 300–1600 m/z at 4,000 amu/s (with not trapping in Q0 and Dynamic fill time) with an ion spray voltage of 3300 applied to a Picotip FS360-75-15-N with ion spray gas (nitrogen). Precursor ions were collided in Q2 using rolling collision energy (maximum allowed CE = 80). Enhanced product ion scans (MS/MS) were performed over a mass range of 100–1700 m/z at 1000 amu/sec, and collision voltages were determined dynamically. All precursor ion mass/charge ratios were confirmed with an Enhance Resolution scan. Protein identification was done by using the Mascot algorithm (http://www.matrixscience.com), with the SwissProt database; the search parameters included trypsin digestion, MS/MS ion search, monoisotropic mass application, protein mass unrestricted, peptide mass tolerance of ±1.2 Da, fragment mass tolerance of ±0.6 Da and Max Missed Cleavages of 1.

The data associated with this manuscript may be downloaded from ProteomeCommons.org Tranche using the following hash: hAB36ZvUMqsCgDBALN0mJQ4dts+h4YiAIk5ZSasBjaG2TKhIztzBenjpxYlZaoeq41YheUt9ahhLnC2iPCGKy0SDsGwAAAAAAAAntw =  = .

## Supporting Information

Figure S1 An analysis was conducted combining the obtained signaling and transcriptional regulation pathways; the edges indicate the regulatory or hierarchical relationship, and the nodes indicate the pathway. The colors denote each of the hallmarks of cancer, with the two most representative hallmarks indicated per node.
(TIFF)

File S1 **Supporting Tables S1–S12.** Table S1: Enriched gene ontology level 3 categories of biological processes of total expression analysis. Table S2: Enriched gene ontology level 3 categories of biological processes of over-expressed genes. Table S3: Enriched gene ontology level 3 categories of biological processes of over-expressed-TFs networks. Table S4: Enriched gene ontology level 3 categories of biological processes of under-expressed-TFs networks. Table S5: Enriched PID pathway-based sets of over-expressed transcripts. Table S6: Enriched PID pathway-based sets of sub-expressed transcripts. Table S7: list of over and under expressed transcripts members of the regulatory network “Direct effectors of p53”. Table S8: Enriched KEGG pathway-based sets of under-expressed transcripts. Table S9: Phosphoproteins relationship according to which replicates were identified. Table S10: Enriched gene ontology level 3 categories of biological processes of identified phosphoproteins. Table S11: Enriched PID pathway-based sets of identified phosphoproteins. Table S12: Enriched KEGG pathway-based sets of identified phosphoproteins.
(PDF)

Spreadsheet S1 Statistical data of the RNA-Seq analysis shows the full dataset, and the data with significant differential expression.
(XML)
