# Correction: Analysis and Prediction of Pathways in HeLa Cells by Integrating Biological Levels of Organization with Systems-Biology Approaches

**DOI:** 10.1371/annotation/b212d5e5-7c4b-48c1-80ff-ba0d4ce87cfc

**Published:** 2013-07-01

**Authors:** Juan Carlos Higareda-Almaraz, Ilse A. Valtierra-Gutiérrez, Magdalena Hernandez-Ortiz, Sandra Contreras, Erika Hernandez, Sergio Encarnacion

The name of the sixth author was given incorrectly. The correct name is: Sergio Encarnación-Guevara. The correct abbreviation in Contributions is: SEG. 

