# Supplementary figures and images for: Analysis and Prediction of Pathways in HeLa Cells by Integrating Biological Levels of Organization with Systems-Biology Approaches
Source: PLoS One. 2013 Jun 10;8(6):e65433. doi: 10.1371/journal.pone.0065433 (PMC3680226; doi:10.1371/journal.pone.0065433)

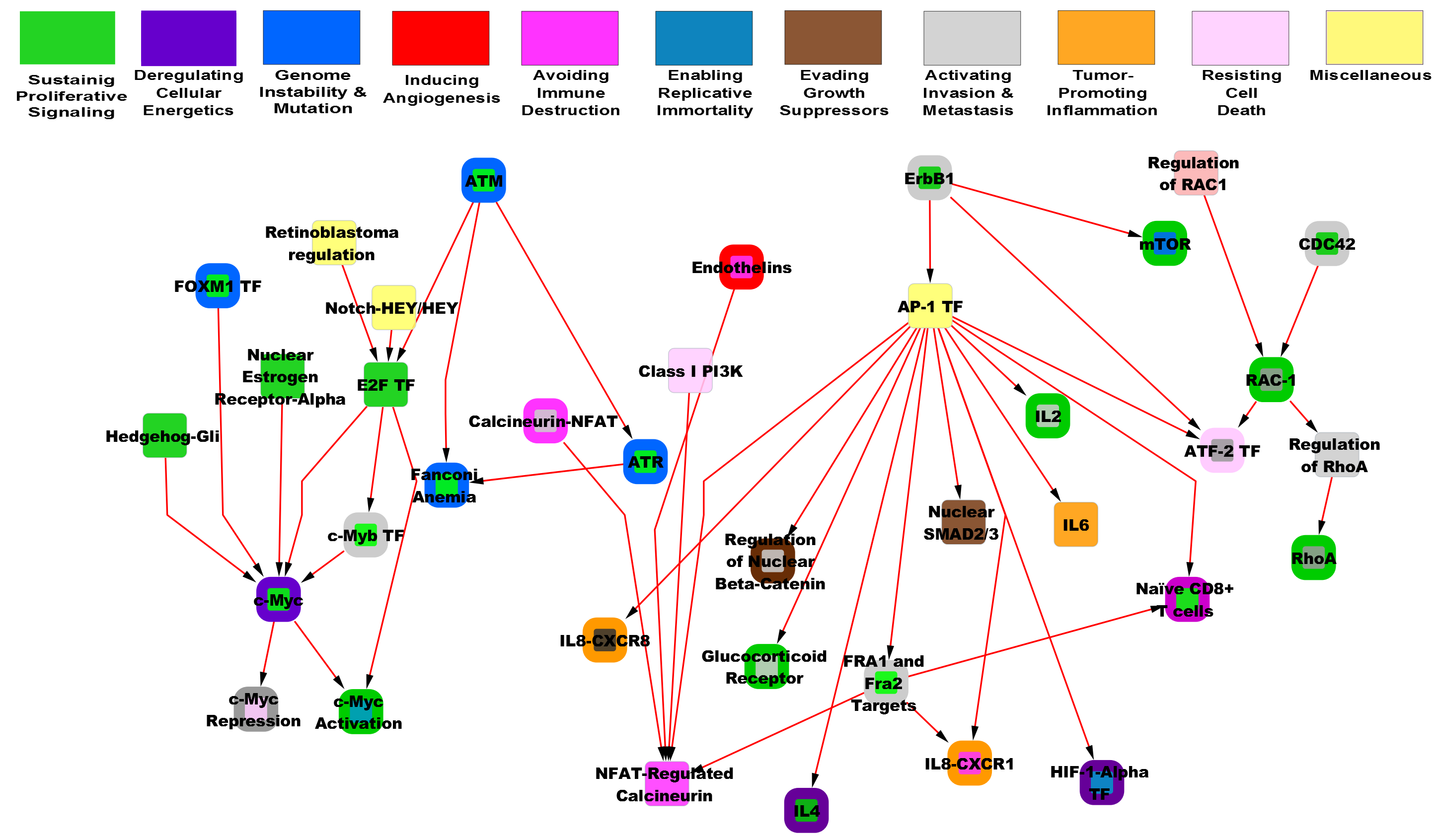

Supplement: Figure S1 — An analysis was conducted combining the obtained signaling and transcriptional regulation pathways; the edges indicate the regulatory or hierarchical relationship, and the nodes indicate the pathway. The colors denote each of the hallmarks of cancer, with the two most representative hallmarks indicated per node. (TIFF) [file pone.0065433.s001.tiff]
