# Supplementary material for: Analysis and Prediction of Pathways in HeLa Cells by Integrating Biological Levels of Organization with Systems-Biology Approaches
Source: PLoS One. 2013 Jun 10;8(6):e65433. doi: 10.1371/journal.pone.0065433 (PMC3680226; doi:10.1371/journal.pone.0065433)
Supplement: File S1 — Supporting Tables S1–S12. Table S1: Enriched gene ontology level 3 categories of biological processes of total expression analysis. Table S2: Enriched gene ontology level 3 categories of biological processes of over-expressed genes. Table S3: Enriched gene ontology level 3 categories of biological processes of over-expressed-TFs networks. Table S4: Enriched gene ontology level 3 categories of biological processes of under-expressed-TFs networks. Table S5: Enriched PID pathway-based sets of over-expressed transcripts. Table S6: Enriched PID pathway-based sets of sub-expressed transcripts. Table S7: list of over and under expressed transcripts members of the regulatory network “Direct effectors of p53”. Table S8: Enriched KEGG pathway-based sets of under-expressed transcripts. Table S9: Phosphoproteins relationship according to which replicates were identified. Table S10: Enriched gene ontology level 3 categories of biological processes of identified phosphoproteins. Table S11: Enriched PID pathway-based sets of identified phosphoproteins. Table S12: Enriched KEGG pathway-based sets of identified phosphoproteins. (PDF) [file pone.0065433.s002.pdf]

Analysis and prediction of pathways in HeLa cells by integrating biological levels of organization with systems-biology approaches

Supplemental Material

Location	Pathway size	Genes present
GO:0005737 cytoplasm	8475	8253 (97.4%)
GO:0044444 cytoplasmic part	6092	5961 (97.9%)
GO:0043231 intracellular membrane-bounded organelle	8980	8704 (97.0%)
GO:0070013 intracellular organelle lumen	2668	2621 (98.3%)
GO:0044428 nuclear part	2594	2543 (98.1%)
GO:0005626 insoluble fraction	904	896 (99.1%)
GO:0043232 intracellular non-membrane-bounded organelle	3310	3225 (97.5%)
GO:0031967 organelle envelope	751	740 (98.5%)
GO:0044459 plasma membrane part	1958	1908 (97.4%)
GO:0005625 soluble fraction	395	392 (99.2%)
GO:0044429 mitochondrial part	705	694 (98.4%)
GO:0044430 cytoskeletal part	1153	1127 (97.8%)
GO:0044432 endoplasmic reticulum part	770	756 (98.2%)
GO:0044431 Golgi apparatus part	597	587 (98.3%)
GO:0043005 neuron projection	522	514 (98.5%)
GO:0031988 membrane-bounded vesicle	758	743 (98.0%)
GO:0031410 cytoplasmic vesicle	784	768 (98.0%)
GO:0042175 nuclear outer membrane-endoplasmic reticulum membrane network	697	683 (98.0%)
GO:0044437 vacuolar part	172	171 (99.4%)
GO:0031300 intrinsic to organelle membrane	167	166 (99.4%)
GO:0044440 endosomal part	275	271 (98.5%)
GO:0016585 chromatin remodeling complex	109	109 (100.0%)

Table S1 Enriched gene ontology level 3 categories of biological processes of total expression analysis. Set size refers to the number of entities that have a UniProt ID as stated in the corresponding GO category at the ConsensusPathDB site. The number of candidates contained refers to amount of proteins identified in this study that appear as part of the GO category. P-values are calculated according to a hypergeometric test; q-values represent p-values corrected for multiple testing using the false discovery rate method. It is worth noting that the expressions of each GO term fulfill at least 97% of the total reported for each term.

Gene Ontology term	Set size	Candidates contained	P-value	Q-value
GO:0022403 cell cycle phase	772	309 (40.0%)	1.29E-53	7.15E-51
GO:0000278 mitotic cell cycle	687	261 (38.0%)	2.21E-40	6.12E-38
GO:0006139 nucleobase, nucleoside, nucleotide and nucleic acid metabolic process	4958	1120 (22.6%)	8.03E-32	1.48E-29
GO:0034641 cellular nitrogen compound metabolic process	5373	1194 (22.2%)	3.38E-31	4.68E-29
GO:0071842 cellular component organization at cellular level	2857	704 (24.7%)	4.98E-29	5.52E-27
GO:0044260 cellular macromolecule metabolic process	6149	1322 (21.5%)	2.01E-28	1.85E-26
GO:0033554 cellular response to stress	957	262 (27.4%)	2.86E-16	2.26E-14
GO:0051321 meiotic cell cycle	138	62 (44.9%)	2.48E-14	1.72E-12
GO:0007050 cell cycle arrest	335	113 (33.7%)	9.40E-14	5.79E-12
GO:0009059 macromolecule biosynthetic process	3775	798 (21.2%)	4.45E-13	2.46E-11
GO:0044249 cellular biosynthetic process	4603	949 (20.6%)	5.99E-13	3.02E-11
GO:0043933 macromolecular complex subunit organization	1008	254 (25.2%)	1.66E-11	7.65E-10
GO:0010467 gene expression	3914	806 (20.6%)	1.26E-10	5.37E-09
GO:0019222 regulation of metabolic process	4485	907 (20.2%)	3.22E-10	1.28E-08
GO:0000226 microtubule cytoskeleton organization	215	73 (34.0%)	1.60E-09	5.89E-08
GO:0000086 G2/M transition of mitotic cell cycle	135	52 (38.5%)	2.72E-09	9.13E-08
GO:0022607 cellular component assembly	1282	299 (23.3%)	2.80E-09	9.13E-08
GO:0048519 negative regulation of biological process	2583	548 (21.2%)	4.57E-09	1.41E-07
GO:0050794 regulation of cellular process	7306	1393 (19.1%)	1.21E-08	3.53E-07
GO:0000819 sister chromatid segregation	45	24 (53.3%)	3.54E-08	9.82E-07
GO:0007062 sister chromatid cohesion	26	17 (65.4%)	6.04E-08	1.59E-06
GO:0007091 mitotic metaphase/anaphase transition	43	22 (51.2%)	3.45E-07	8.68E-06
GO:0000083 regulation of transcription involved in G1/S phase of mitotic cell cycle	21	14 (66.7%)	6.49E-07	1.53E-05
GO:0007131 reciprocal meiotic recombination	35	19 (54.3%)	6.62E-07	1.53E-05
GO:0043412 macromolecule modification	2212	458 (20.7%)	2.16E-06	4.79E-05
GO:0000082 G1/S transition of mitotic cell cycle	169	53 (31.4%)	4.13E-06	8.80E-05
GO:0006725 cellular aromatic compound metabolic process	223	65 (29.1%)	5.88E-06	0.000121
GO:0007098 centrosome cycle	31	16 (51.6%)	1.20E-05	0.000238
GO:0050658 RNA transport	127	41 (32.3%)	2.27E-05	0.000435
GO:0006403 RNA localization	132	42 (31.8%)	2.69E-05	0.000483
GO:0015931 nucleobase, nucleoside, nucleotide and nucleic acid transport	149	46 (30.9%)	2.70E-05	0.000483
GO:0000216 M/G1 transition of mitotic cell cycle	79	28 (35.4%)	7.04E-05	0.00122

Table S2 Enriched gene ontology level 3 categories of biological processes of over-expressed genes. Set size refers to the number of entities that have a UniProt ID as stated in the corresponding GO category at the ConsensusPathDB site. The number of candidates contained refers to amount of proteins identified in this study that appear as part of the GO category. P-values are calculated according to a hypergeometric test; q-values represent p-values corrected for multiple testing using the false discovery rate method.

Gene Ontology term	Set size	Candidates contained	P-value	Q-value
GO:0022403 cell cycle phase	772	257 (33.3%)	5.33E-49	2.86E-46
GO:0034641 cellular nitrogen compound metabolic process	5373	1002 (18.7%)	7.02E-43	1.88E-40
GO:0006139 nucleobase, nucleoside, nucleotide and nucleic acid metabolic process	4958	936 (18.9%)	2.23E-41	3.98E-39
GO:0044260 cellular macromolecule metabolic process	6149	1098 (17.9%)	6.4E-39	7.59E-37
GO:0000278 mitotic cell cycle	687	220 (32.0%)	7.08E-39	7.59E-37
GO:0071842 cellular component organization at cellular level	2857	602 (21.1%)	1.04E-37	9.33E-36
GO:0044249 cellular biosynthetic process	4603	809 (17.6%)	3.4E-23	2.6E-21
GO:0033554 cellular response to stress	957	234 (24.5%)	2.68E-22	1.8E-20
GO:0009059 macromolecule biosynthetic process	3775	669 (17.7%)	2.83E-19	1.68E-17
GO:0019222 regulation of metabolic process	4485	766 (17.1%)	4.77E-18	2.56E-16
GO:0050794 regulation of cellular process	7306	1156 (15.8%)	1.45E-17	7.08E-16
GO:0043933 macromolecular complex subunit organization	1008	225 (22.4%)	1.81E-16	8.08E-15
GO:0010467 gene expression	3914	667 (17.1%)	4.19E-15	1.73E-13
GO:0007050 cell cycle arrest	335	98 (29.3%)	6.28E-15	2.4E-13
GO:0048519 negative regulation of biological process	2583	469 (18.2%)	8.27E-15	2.95E-13
GO:0022607 cellular component assembly	1282	258 (20.1%)	5.03E-13	1.68E-11
GO:0051321 meiotic cell cycle	138	51 (37.0%)	1.49E-12	4.69E-11
GO:0000086 G2/M transition of mitotic cell cycle	135	49 (36.3%)	8.75E-12	2.6E-10
GO:0048518 positive regulation of biological process	2857	493 (17.3%)	1.03E-11	2.92E-10
GO:0043412 macromolecule modification	2212	384 (17.4%)	1.8E-09	4.84E-08
GO:0042592 homeostatic process	996	196 (19.7%)	2.94E-09	0.000000075
GO:0048731 system development	3031	501 (16.5%)	5.11E-09	0.000000125
GO:0000819 sister chromatid segregation	45	22 (48.9%)	8.2E-09	0.000000191
GO:0007131 reciprocal meiotic recombination	35	19 (54.3%)	9.23E-09	0.000000206
GO:0019538 protein metabolic process	3414	553 (16.2%)	1.36E-08	0.000000291
GO:0048513 organ development	2079	358 (17.2%)	1.75E-08	0.00000036
GO:0000226 microtubule cytoskeleton organization	215	59 (27.4%)	2.09E-08	0.000000415
GO:0007091 mitotic metaphase/anaphase transition	43	20 (46.5%)	0.000000113	0.00000216
GO:0030154 cell differentiation	2273	379 (16.7%)	0.000000256	0.00000473
GO:0009725 response to hormone stimulus	660	132 (20.0%)	0.000000492	0.00000879
GO:0051128 regulation of cellular component organization	935	175 (18.7%)	0.000000733	0.0000127
GO:0010033 response to organic substance	1394	245 (17.6%)	0.000000895	0.000015
GO:0001666 response to hypoxia	203	52 (25.6%)	0.00000143	0.0000233
GO:0070482 response to oxygen levels	221	55 (24.9%)	0.00000191	0.0000301
GO:0006793 phosphorus metabolic process	1306	228 (17.5%)	0.00000363	0.0000556
GO:0070887 cellular response to chemical stimulus	1112	197 (17.7%)	0.00000674	0.0001
GO:0050793 regulation of developmental process	1066	189 (17.7%)	0.00000994	0.000144
GO:0070271 protein complex biogenesis	604	117 (19.4%)	0.0000106	0.000149
GO:0006725 cellular aromatic compound metabolic process	223	53 (23.8%)	0.0000122	0.000167

GO:0000082 G1/S transition of mitotic cell cycle	169	43 (25.4%)	0.0000135	0.000181
GO:0007599 hemostasis	512	101 (19.7%)	0.0000194	0.000254
GO:0009792 embryo development ending in birth or egg hatching	399	82 (20.6%)	0.000026	0.000331
GO:0007596 blood coagulation	508	99 (19.5%)	0.0000387	0.000483
GO:0010035 response to inorganic substance	332	70 (21.1%)	0.0000424	0.000507
GO:0042493 response to drug	338	71 (21.0%)	0.0000425	0.000507
GO:0007062 sister chromatid cohesion	26	12 (46.2%)	0.0000445	0.000518
GO:0012501 programmed cell death	1390	233 (16.8%)	0.0000489	0.000557
GO:0009611 response to wounding	1017	177 (17.4%)	0.0000554	0.000619
GO:0007098 centrosome cycle	31	13 (41.9%)	0.0000738	0.000808
GO:0050658 RNA transport	127	33 (26.0%)	0.0000836	0.000896
GO:0000083 regulation of transcription involved in G1/S phase of mitotic cell cycle	21	10 (47.6%)	0.000141	0.00148
GO:0000216 M/G1 transition of mitotic cell cycle	79	23 (29.1%)	0.000158	0.0016
GO:0007052 mitotic spindle organization	25	11 (44.0%)	0.000158	0.0016
GO:0006403 RNA localization	132	33 (25.0%)	0.000185	0.00184
GO:0015931 nucleobase, nucleoside, nucleotide and nucleic acid transport	149	36 (24.2%)	0.000202	0.00197
GO:0001568 blood vessel development	386	76 (19.7%)	0.000212	0.00202
GO:0048514 blood vessel morphogenesis	339	67 (19.8%)	0.000436	0.0041
GO:0005975 carbohydrate metabolic process	646	115 (17.8%)	0.000464	0.00429
GO:0051239 regulation of multicellular organismal process	1402	227 (16.2%)	0.000497	0.00451
GO:0006959 humoral immune response	112	28 (25.0%)	0.00055	0.00491
GO:0035295 tube development	331	65 (19.6%)	0.000628	0.00552
GO:0048468 cell development	1211	198 (16.4%)	0.000694	0.006
GO:0006979 response to oxidative stress	223	47 (21.1%)	0.000724	0.00616
GO:0044093 positive regulation of molecular function	982	164 (16.7%)	0.000772	0.00646
GO:0001525 angiogenesis	285	57 (20.0%)	0.000834	0.00688
GO:0044283 small molecule biosynthetic process	666	116 (17.4%)	0.000951	0.00772
GO:0006091 generation of precursor metabolites and energy	427	79 (18.5%)	0.00113	0.009
GO:0009057 macromolecule catabolic process	715	123 (17.2%)	0.00115	0.009
GO:0002237 response to molecule of bacterial origin	186	40 (21.5%)	0.00116	0.009
GO:0035821 modification of morphology or physiology of other organism	35	12 (34.3%)	0.00122	0.00936
GO:0042023 DNA endoreduplication	5	4 (80.0%)	0.00137	0.0103
GO:0048469 cell maturation	91	23 (25.3%)	0.00141	0.0105
GO:0007584 response to nutrient	225	46 (20.4%)	0.00159	0.0115
GO:0009314 response to radiation	268	53 (19.8%)	0.00161	0.0115
GO:0071495 cellular response to endogenous stimulus	381	71 (18.6%)	0.00161	0.0115
GO:0007165 signal transduction	3729	548 (14.7%)	0.00174	0.0122
GO:0040008 regulation of growth	440	80 (18.2%)	0.00176	0.0122
GO:0021915 neural tube development	99	24 (24.2%)	0.00207	0.0142
GO:0048659 smooth muscle cell proliferation	62	17 (27.4%)	0.00225	0.0152
GO:0009636 response to toxin	95	23 (24.2%)	0.00258	0.0173

GO:0051303 establishment of chromosome localization	20	8 (40.0%)	0.00266	0.0174
GO:0060323 head morphogenesis	20	8 (40.0%)	0.00266	0.0174
GO:0042558 pteridine-containing compound metabolic process	34	11 (32.4%)	0.00327	0.0211
GO:0046677 response to antibiotic	30	10 (33.3%)	0.00389	0.0248
GO:0046148 pigment biosynthetic process	45	13 (28.9%)	0.00438	0.0276
GO:0009991 response to extracellular stimulus	351	64 (18.2%)	0.00446	0.0278
GO:0009887 organ morphogenesis	660	110 (16.7%)	0.00568	0.035
GO:0051702 interaction with symbiont	18	7 (38.9%)	0.00589	0.0356
GO:0045137 development of primary sexual characteristics	203	40 (19.7%)	0.00597	0.0356
GO:0046483 heterocycle metabolic process	1019	162 (15.9%)	0.00611	0.0356
GO:0001556 oocyte maturation	14	6 (42.9%)	0.00618	0.0356
GO:0010573 vascular endothelial growth factor production	14	6 (42.9%)	0.00618	0.0356
GO:0045132 meiotic chromosome segregation	14	6 (42.9%)	0.00618	0.0356
GO:0006066 alcohol metabolic process	521	89 (17.1%)	0.00628	0.0358
GO:0055086 nucleobase, nucleoside and nucleotide metabolic process	911	146 (16.0%)	0.00674	0.038
GO:0031647 regulation of protein stability	74	18 (24.3%)	0.00682	0.0381
GO:0007548 sex differentiation	230	44 (19.1%)	0.00707	0.0391
GO:0032844 regulation of homeostatic process	181	36 (19.9%)	0.00754	0.0412
GO:0044248 cellular catabolic process	1483	227 (15.3%)	0.00838	0.0453
GO:0007099 centriole replication	11	5 (45.5%)	0.00932	0.05
GO:0048729 tissue morphogenesis	350	62 (17.7%)	0.00953	0.0506
GO:0042180 cellular ketone metabolic process	802	129 (16.1%)	0.00963	0.0506
GO:0044403 symbiosis, encompassing mutualism through parasitism	88	20 (22.7%)	0.00991	0.0516

Table S3 Enriched gene ontology level 3 categories of biological processes of over-expressed-TFs networks. Set size refers to the number of entities that have a UniProt ID as stated in the corresponding GO category at the ConsensusPathDB site. The number of candidates contained refers to amount of proteins identified in this study that appear as part of the GO category. P-values are calculated according to a hypergeometric test; q-values represent p-values corrected for multiple testing using the false discovery rate method.

gene ontology term	set size	candidates contained	p-value	q-value
GO:0048731 system development	3031	415 (13.7%)	1.36E-26	7.11E-24
GO:0009888 tissue development	1010	181 (17.9%)	4.44E-23	1.16E-20
GO:0007165 signal transduction	3729	459 (12.3%)	1.16E-19	2.02E-17
GO:0048513 organ development	2079	291 (14.0%)	2.23E-19	2.92E-17
GO:0030154 cell differentiation	2273	308 (13.6%)	2.14E-18	2.23E-16
GO:0051239 regulation of multicellular organismal process	1402	205 (14.6%)	1.52E-15	1.33E-13
GO:0010033 response to organic substance	1394	201 (14.4%)	1.28E-14	9.56E-13
GO:0032989 cellular component morphogenesis	800	133 (16.6%)	1.96E-14	1.28E-12
GO:0048514 blood vessel morphogenesis	339	74 (21.8%)	2.21E-14	1.29E-12
GO:0009611 response to wounding	1017	158 (15.5%)	2.48E-14	1.3E-12
GO:0001525 angiogenesis	285	65 (22.8%)	1.01E-13	4.8E-12
GO:0051128 regulation of cellular component organization	935	146 (15.6%)	1.75E-13	7.29E-12
GO:0070887 cellular response to chemical stimulus	1112	166 (14.9%)	1.81E-13	7.29E-12
GO:0001568 blood vessel development	386	78 (20.2%)	3.31E-13	1.24E-11
GO:0048468 cell development	1211	174 (14.4%)	1.35E-12	4.69E-11
GO:0034329 cell junction assembly	149	42 (28.2%)	1.63E-12	5.34E-11
GO:0048583 regulation of response to stimulus	1870	242 (12.9%)	3.65E-12	1.12E-10
GO:0050794 regulation of cellular process	7306	746 (10.2%)	5.3E-12	1.54E-10
GO:0023051 regulation of signaling	1622	213 (13.1%)	2.39E-11	6.57E-10
GO:0032879 regulation of localization	1009	146 (14.5%)	6.23E-11	1.63E-09
GO:0050793 regulation of developmental process	1066	152 (14.3%)	7.35E-11	1.74E-09
GO:0048518 positive regulation of biological process	2857	334 (11.7%)	7.43E-11	1.74E-09
GO:0006629 lipid metabolic process	1030	148 (14.4%)	7.63E-11	1.74E-09
GO:0048519 negative regulation of biological process	2583	303 (11.7%)	5.29E-10	1.15E-08
GO:0040012 regulation of locomotion	315	61 (19.4%)	7.63E-10	0.000000016
GO:0009887 organ morphogenesis	660	103 (15.6%)	8.16E-10	1.64E-08
GO:0048729 tissue morphogenesis	350	65 (18.6%)	1.28E-09	2.49E-08
GO:0045216 cell-cell junction organization	100	28 (28.0%)	9.7E-09	0.000000181
GO:0046903 secretion	716	103 (14.4%)	6.44E-08	0.00000116
GO:0012501 programmed cell death	1390	174 (12.5%)	6.82E-08	0.00000119
GO:0022612 gland morphogenesis	90	25 (27.8%)	7.07E-08	0.00000119
GO:0030036 actin cytoskeleton organization	337	58 (17.2%)	0.000000159	0.0000026
GO:0035295 tube development	331	57 (17.2%)	0.000000199	0.00000316
GO:0002682 regulation of immune system process	748	103 (13.8%)	0.000000555	0.00000854
GO:0030168 platelet activation	246	45 (18.3%)	0.000000683	0.0000102
GO:0044255 cellular lipid metabolic process	706	98 (13.9%)	0.000000729	0.0000106
GO:0002237 response to molecule of bacterial origin	186	37 (19.9%)	0.000000822	0.0000116
GO:0010035 response to inorganic substance	332	55 (16.6%)	0.00000115	0.0000159
GO:0071842 cellular component organization at cellular level	2857	309 (10.8%)	0.00000125	0.0000168
GO:0061024 membrane organization	552	80 (14.5%)	0.00000145	0.0000185
GO:0070482 response to oxygen levels	221	41 (18.6%)	0.00000145	0.0000185

GO:0008610 lipid biosynthetic process	448	68 (15.2%)	0.00000176	0.0000219
GO:0032612 interleukin-1 production	39	14 (35.9%)	0.00000188	0.0000229
GO:0044093 positive regulation of molecular function	982	125 (12.7%)	0.00000238	0.0000282
GO:0001666 response to hypoxia	203	38 (18.7%)	0.00000278	0.0000323
GO:0044092 negative regulation of molecular function	554	79 (14.3%)	0.00000316	0.0000359
GO:0044087 regulation of cellular component biogenesis	240	42 (17.5%)	0.00000514	0.0000572
GO:0006793 phosphorus metabolic process	1306	156 (11.9%)	0.00000559	0.0000609
GO:0042633 hair cycle	73	19 (26.0%)	0.00000737	0.0000787
GO:0006897 endocytosis	303	49 (16.2%)	0.00000861	0.0000883
GO:0010324 membrane invagination	303	49 (16.2%)	0.00000861	0.0000883
GO:0022404 molting cycle process	69	18 (26.1%)	0.0000123	0.000124
GO:0007599 hemostasis	512	72 (14.1%)	0.0000139	0.000137
GO:0006952 defense response	984	121 (12.3%)	0.000018	0.000175
GO:0035239 tube morphogenesis	220	38 (17.3%)	0.0000193	0.000183
GO:0007596 blood coagulation	508	71 (14.0%)	0.0000196	0.000183
GO:0016337 cell-cell adhesion	330	51 (15.5%)	0.0000205	0.000188
GO:0006066 alcohol metabolic process	521	72 (13.8%)	0.0000251	0.000226
GO:0031589 cell-substrate adhesion	169	31 (18.3%)	0.0000336	0.000297
GO:0061061 muscle structure development	363	54 (14.9%)	0.0000352	0.000307
GO:0090132 epithelium migration	18	8 (44.4%)	0.0000534	0.000451
GO:0032844 regulation of homeostatic process	181	32 (17.7%)	0.0000535	0.000451
GO:0048608 reproductive structure development	224	37 (16.5%)	0.0000663	0.000544
GO:0009725 response to hormone stimulus	660	85 (12.9%)	0.0000665	0.000544
GO:0043412 macromolecule modification	2212	236 (10.7%)	0.0000779	0.000618
GO:0042493 response to drug	338	50 (14.8%)	0.000078	0.000618
GO:0022607 cellular component assembly	1282	146 (11.4%)	0.000118	0.000918
GO:0060560 developmental growth involved in morphogenesis	97	20 (20.6%)	0.000158	0.00122
GO:0071706 tumor necrosis factor superfamily cytokine production	62	15 (24.2%)	0.000162	0.00122
GO:0010876 lipid localization	200	33 (16.5%)	0.000164	0.00122
GO:0045087 innate immune response	440	60 (13.6%)	0.000168	0.00124
GO:0044259 multicellular organismal macromolecule metabolic process	50	13 (26.0%)	0.000203	0.00147
GO:0032943 mononuclear cell proliferation	153	27 (17.6%)	0.000205	0.00147
GO:0042592 homeostatic process	996	116 (11.6%)	0.00026	0.00184
GO:0006979 response to oxidative stress	223	35 (15.7%)	0.000286	0.00197
GO:0007620 copulation	22	8 (36.4%)	0.000286	0.00197
GO:0006887 exocytosis	241	37 (15.4%)	0.000304	0.00207
GO:0060056 mammary gland involution	13	6 (46.2%)	0.000377	0.00253
GO:0048839 inner ear development	111	21 (18.9%)	0.000384	0.00254
GO:0061138 morphogenesis of a branching epithelium	136	24 (17.6%)	0.00045	0.00294
GO:0001935 endothelial cell proliferation	68	15 (22.1%)	0.000478	0.00308
GO:0040008 regulation of growth	440	58 (13.2%)	0.000514	0.00328
GO:0019538 protein metabolic process	3414	339 (9.9%)	0.000527	0.00332

GO:0032535 regulation of cellular component size	146	25 (17.1%)	0.000552	0.00342
GO:0048598 embryonic morphogenesis	385	52 (13.5%)	0.000556	0.00342
GO:0055082 cellular chemical homeostasis	510	65 (12.7%)	0.000613	0.00373
GO:0031099 regeneration	115	21 (18.3%)	0.000627	0.00377
GO:0007584 response to nutrient	225	34 (15.1%)	0.000699	0.00415
GO:0003179 heart valve morphogenesis	10	5 (50.0%)	0.000767	0.00442
GO:0071495 cellular response to endogenous stimulus	381	51 (13.4%)	0.000768	0.00442
GO:0016486 peptide hormone processing	25	8 (32.0%)	0.00077	0.00442
GO:0090066 regulation of anatomical structure size	245	36 (14.7%)	0.000838	0.00476
GO:0030728 ovulation	20	7 (35.0%)	0.000903	0.00508
GO:0009612 response to mechanical stimulus	111	20 (18.0%)	0.000994	0.00553
GO:0031069 hair follicle morphogenesis	26	8 (30.8%)	0.00103	0.00561
GO:0008038 neuron recognition	26	8 (30.8%)	0.00103	0.00561
GO:0044282 small molecule catabolic process	800	93 (11.6%)	0.0011	0.00593
GO:0048645 organ formation	39	10 (25.6%)	0.00122	0.0065
GO:0003170 heart valve development	11	5 (45.5%)	0.00131	0.00687
GO:0060349 bone morphogenesis	46	11 (23.9%)	0.00131	0.00687
GO:0030595 leukocyte chemotaxis	90	17 (18.9%)	0.00135	0.00695
GO:0071695 anatomical structure maturation	27	8 (29.6%)	0.00136	0.00695
GO:0071496 cellular response to external stimulus	190	29 (15.3%)	0.00142	0.00722
GO:0071702 organic substance transport	498	62 (12.4%)	0.00145	0.0073
GO:0048546 digestive tract morphogenesis	40	10 (25.0%)	0.0015	0.00748
GO:0051098 regulation of binding	124	21 (16.9%)	0.0017	0.00835
GO:0009595 detection of biotic stimulus	22	7 (31.8%)	0.00171	0.00835
GO:0045137 development of primary sexual characteristics	203	30 (14.8%)	0.00199	0.00955
GO:0008104 protein localization	1252	135 (10.8%)	0.00199	0.00955
GO:0070271 protein complex biogenesis	604	72 (11.9%)	0.00203	0.00966
GO:0048286 lung alveolus development	35	9 (25.7%)	0.00207	0.00972
GO:0009991 response to extracellular stimulus	351	46 (13.1%)	0.00208	0.00972
GO:0046849 bone remodeling	42	10 (23.8%)	0.00223	0.0103
GO:0021675 nerve development	49	11 (22.4%)	0.00227	0.0104
GO:0002568 somatic diversification of T cell receptor genes	4	3 (75.0%)	0.00229	0.0104
GO:0003012 muscle system process	252	35 (13.9%)	0.00261	0.0118
GO:0001946 lymphangiogenesis	8	4 (50.0%)	0.00274	0.0122
GO:0070997 neuron death	146	23 (15.8%)	0.0028	0.0124
GO:0016042 lipid catabolic process	226	32 (14.2%)	0.00287	0.0126
GO:0045104 intermediate filament cytoskeleton organization	24	7 (29.2%)	0.00298	0.013
GO:0032602 chemokine production	37	9 (24.3%)	0.00313	0.0135
GO:0001945 lymph vessel development	13	5 (38.5%)	0.00316	0.0135
GO:0045165 cell fate commitment	174	26 (14.9%)	0.00328	0.0139
GO:0003002 regionalization	247	34 (13.8%)	0.00344	0.0145
GO:0009792 embryo development ending in birth or egg hatching	399	50 (12.5%)	0.00348	0.0146

GO:0002089	lens morphogenesis in camera-type eye	19	6 (31.6%)	0.00382	0.0159
GO:0048754	branching morphogenesis of a tube	124	20 (16.1%)	0.00388	0.016
GO:0003013	circulatory system process	278	37 (13.3%)	0.00417	0.017
GO:0044248	cellular catabolic process	1483	154 (10.4%)	0.00436	0.0177
GO:0002088	lens development in camera-type eye	46	10 (21.7%)	0.00453	0.0181
GO:0042403	thyroid hormone metabolic process	14	5 (35.7%)	0.00457	0.0181
GO:0009415	response to water	9	4 (44.4%)	0.0046	0.0181
GO:0042640	anagen	9	4 (44.4%)	0.0046	0.0181
GO:0006082	organic acid metabolic process	799	89 (11.1%)	0.00472	0.0184
GO:0010817	regulation of hormone levels	357	45 (12.6%)	0.00483	0.0187
GO:0021536	diencephalon development	54	11 (20.4%)	0.00504	0.0194
GO:0030900	forebrain development	226	31 (13.7%)	0.00533	0.0202
GO:0048659	smooth muscle cell proliferation	62	12 (19.4%)	0.00537	0.0202
GO:0016197	endosome transport	119	19 (16.0%)	0.00538	0.0202
GO:0060512	prostate gland morphogenesis	27	7 (25.9%)	0.00611	0.0228
GO:0060343	trabecula formation	15	5 (33.3%)	0.00638	0.0237
GO:0010259	multicellular organismal aging	21	6 (28.6%)	0.0066	0.0243
GO:0007548	sex differentiation	230	31 (13.5%)	0.00687	0.0251
GO:0060008	Sertoli cell differentiation	10	4 (40.0%)	0.00716	0.0251
GO:0021984	adenohypophysis development	10	4 (40.0%)	0.00716	0.0251
GO:0001550	ovarian cumulus expansion	2	2 (100.0%)	0.0072	0.0251
GO:0002238	response to molecule of fungal origin	2	2 (100.0%)	0.0072	0.0251
GO:0006788	heme oxidation	2	2 (100.0%)	0.0072	0.0251
GO:0045229	external encapsulating structure organization	2	2 (100.0%)	0.0072	0.0251
GO:0042335	cuticle development	2	2 (100.0%)	0.0072	0.0251
GO:0060711	labyrinthine layer development	28	7 (25.0%)	0.00756	0.0262
GO:0072593	reactive oxygen species metabolic process	98	16 (16.3%)	0.00818	0.0281
GO:0072006	nephron development	50	10 (20.0%)	0.00838	0.0287
GO:0061383	trabecula morphogenesis	16	5 (31.2%)	0.00864	0.0292
GO:0006805	xenobiotic metabolic process	142	21 (14.8%)	0.00865	0.0292
GO:0048588	developmental cell growth	74	13 (17.6%)	0.00891	0.0299
GO:0035019	somatic stem cell maintenance	29	7 (24.1%)	0.00926	0.0308
GO:0035148	tube formation	83	14 (16.9%)	0.00973	0.0322

Table S4 Enriched gene ontology level 3 categories of biological processes of under-expressed-TFs networks. Set size refers to the number of entities that have a UniProt ID as stated in the corresponding GO category at the ConsensusPathDB site. The number of candidates contained refers to amount of proteins identified in this study that appear as part of the GO category. P-values are calculated according to a hypergeometric test; q-values represent p-values corrected for multiple testing using the false discovery rate method.

**Pathway name**	**Set size**	**Candidates contained**	**P-value**	**Q-value**
Fanconi anemia pathway	47	32	4.66E-10	6.80E-08
AP-1 transcription factor network	70	26	2.98E-08	1.45E-06
ATR signaling pathway	37	26	2.98E-08	1.45E-06
E2F transcription factor network	77	38	8.05E-08	2.94E-06
PLK1 signaling events	44	24	1.19E-07	3.48E-06
Regulation of Telomerase	73	23	2.38E-07	5.80E-06
Direct p53 effectors	144	21	9.54E-07	1.74E-05
FOXM1 transcription factor network	42	21	9.54E-07	1.74E-05
Beta1 integrin cell surface interactions	66	20	1.91E-06	3.09E-05
Aurora B signaling	43	19	3.81E-06	5.06E-05
Regulation of nuclear SMAD2/3 signaling	78	19	3.81E-06	5.06E-05
C-MYB transcription factor network	85	18	7.63E-06	7.43E-05
ATF-2 transcription factor network	61	18	7.63E-06	7.43E-05
Regulation of retinoblastoma protein	66	18	7.63E-06	7.43E-05
PDGFR-beta signaling pathway	130	18	7.63E-06	7.43E-05
Validated targets of C-MYC transcriptional activation	87	26	8.80E-06	8.03E-05
HIF-1-alpha transcription factor network	66	17	1.53E-05	0.000117
BARD1 signaling events	31	17	1.53E-05	0.000117
p73 transcription factor network	79	17	1.53E-05	0.000117
Validated nuclear estrogen receptor alpha network	66	16	3.05E-05	0.000171
Caspase cascade in apoptosis	53	16	3.05E-05	0.000171
ATM pathway	35	16	3.05E-05	0.000171
Validated targets of C-MYC transcriptional repression	75	16	3.05E-05	0.000171
Coregulation of Androgen receptor activity	66	16	3.05E-05	0.000171
Integrins in angiogenesis	65	16	3.05E-05	0.000171
Glucocorticoid receptor regulatory network	80	16	3.05E-05	0.000171
Signaling events mediated by HDAC Class III	40	17	0.000117	0.000575
ErbB1 downstream signaling	108	14	0.000122	0.000575
p53 pathway	59	14	0.000122	0.000575
mTOR signaling pathway	65	14	0.000122	0.000575
IL4-mediated signaling events	66	14	0.000122	0.000575
Validated transcriptional targets of TAp63 isoforms	55	13	0.000244	0.00105
Syndecan-1-mediated signaling events	44	13	0.000244	0.00105
Regulation of Androgen receptor activity	51	13	0.000244	0.00105
HIF-2-alpha transcription factor network	36	12	0.000488	0.00183
Validated transcriptional targets of AP1 family members Fra1 and Fra2	37	12	0.000488	0.00183
RhoA signaling pathway	47	12	0.000488	0.00183
Beta3 integrin cell surface interactions	44	12	0.000488	0.00183
RAC1 signaling pathway	54	12	0.000488	0.00183

Regulation of RhoA activity	47	11	0.000977	0.00297
Notch-mediated HES/HEY network	48	11	0.000977	0.00297
CXCR4-mediated signaling events	89	11	0.000977	0.00297
Validated transcriptional targets of deltaNp63 isoforms	47	11	0.000977	0.00297
Downstream signaling in naïve CD8+ T cells	68	11	0.000977	0.00297
IL2-mediated signaling events	56	11	0.000977	0.00297
FoxO family signaling	50	11	0.000977	0.00297
FOXA2 and FOXA3 transcription factor networks	46	11	0.000977	0.00297
Signaling events mediated by HDAC Class I	57	11	0.000977	0.00297
Endothelins	65	10	0.00195	0.00483
HIV-1 Nef: Negative effector of Fas and TNF-alpha	35	10	0.00195	0.00483
IL8- and CXCR1-mediated signaling events	28	10	0.00195	0.00483
Thromboxane A2 receptor signaling	57	10	0.00195	0.00483
Arf6 trafficking events	50	10	0.00195	0.00483
Role of Calcineurin-dependent NFAT signaling in lymphocytes	57	10	0.00195	0.00483
Calcineurin-regulated NFAT-dependent transcription in lymphocytes	50	10	0.00195	0.00483
CDC42 signaling events	72	10	0.00195	0.00483
Signaling events mediated by PTP1B	53	10	0.00195	0.00483
Signaling events mediated by Hepatocyte Growth Factor Receptor (c-Met)	84	10	0.00195	0.00483
LKB1 signaling events	45	10	0.00195	0.00483
Regulation of nuclear beta catenin signaling and target gene transcription	81	11	0.00384	0.00803
Regulation of RAC1 activity	39	9	0.00391	0.00803
LPA receptor mediated events	63	9	0.00391	0.00803
Signaling events mediated by PRL	24	9	0.00391	0.00803
p75(NTR)-mediated signaling	71	9	0.00391	0.00803
Ceramide signaling pathway	48	9	0.00391	0.00803
IL8- and CXCR2-mediated signaling events	34	9	0.00391	0.00803
Hedgehog signaling events mediated by Gli proteins	49	9	0.00391	0.00803
FGF signaling pathway	50	9	0.00391	0.00803
Neurotrophic factor-mediated Trk receptor signaling	65	9	0.00391	0.00803
IL6-mediated signaling events	48	9	0.00391	0.00803
Class I PI3K signaling events	45	9	0.00391	0.00803
C-MYC pathway	25	8	0.00781	0.0137
FAS (CD95) signaling pathway	35	8	0.00781	0.0137
Aurora A signaling	31	8	0.00781	0.0137
PAR1-mediated thrombin signaling events	44	8	0.00781	0.0137
IL12-mediated signaling events	64	8	0.00781	0.0137
Signaling events mediated by Stem cell factor receptor (c-Kit)	53	8	0.00781	0.0137

EPO signaling pathway	34	8	0.00781	0.0137
RXR and RAR heterodimerization with other nuclear receptor	26	8	0.00781	0.0137
FOXA1 transcription factor network	45	8	0.00781	0.0137
Insulin Pathway	48	8	0.00781	0.0137
Syndecan-4-mediated signaling events	32	8	0.00781	0.0137
Signaling events mediated by VEGFR1 and VEGFR2	72	8	0.00781	0.0137

Table S5 Enriched PID pathway-based sets of over-expressed transcripts. Set size refers to the number of entities that have a Uniprot ID as stated in the corresponding PID pathway-based set at the ConsensusPathDB site. The number of candidates contained refers to amount of proteins which are part of the extended network and appear as part of the pathway. P-values are calculated according to a hypergeometric test; q-values represent p-values corrected for multiple testing using the false discovery rate method.

Pathway name	Set size	Candidates contained	P-value	Q-value
Validated transcriptional targets of deltaNp63 isoforms	47	17 (36.2%)	9.63E-06	0.00177
Validated transcriptional targets of TAp63 isoforms	55	16 (29.6%)	0.000268	0.0247
E-cadherin signaling in the nascent adherens junction	39	12 (31.6%)	0.000823	0.0406
ErbB receptor signaling network	16	7 (43.8%)	0.0012	0.0406
a6b1 and a6b4 Integrin signaling	46	13 (28.9%)	0.00129	0.0406
Posttranslational regulation of adherens junction stability and dissassembly	57	15 (26.8%)	0.00132	0.0406
Endogenous TLR signaling	26	9 (34.6%)	0.00179	0.0469
Direct p53 effectors	144	28 (19.9%)	0.00277	0.0637
Calcineurin-regulated NFAT-dependent transcription in lymphocytes	50	13 (26.0%)	0.00362	0.0739
Nectin adhesion pathway	32	9 (29.0%)	0.00678	0.113
Syndecan-2-mediated signaling events	42	11 (26.2%)	0.00678	0.113
Validated transcriptional targets of AP1 family members Fra1 and Fra2	37	10 (27.0%)	0.00764	0.117
Regulation of nuclear beta catenin signaling and target gene transcription	81	17 (21.2%)	0.00894	0.118
Beta5 beta6 beta7 and beta8 integrin cell surface interactions	17	6 (35.3%)	0.00944	0.118
Cellular roles of Anthrax toxin	22	7 (31.8%)	0.00964	0.118

Table S6 Enriched PID pathway-based sets of sub-expressed transcripts. Set size refers to the number of entities that have a Uniprot ID as stated in the corresponding PID pathway-based set at the ConsensusPathDB site. The number of candidates contained refers to amount of proteins which are part of the extended network and appear as part of the pathway. P-values are calculated according to a hypergeometric test; q-values represent p-values corrected for multiple testing using the false discovery rate method.

Over-expressed transcripts	Folds (log2)		Under-expressed transcripts	Folds (log2)
AIFM2_HUMAN	3.20816		COIA1_HUMAN	-6.30929
KAT2A_HUMAN	2.12567		S10A2_HUMAN	-7.42878
MSH2_HUMAN	2.86183		VDR_HUMAN	-1.50616
LIF_HUMAN	4.00723		PML_HUMAN	-0.824712
DUS5_HUMAN	1.86765		MMP2_HUMAN	-8.4294
BBC3_HUMAN	1.36481		RIR2B_HUMAN	-1.16613
JMY_HUMAN	1.20239		PLK3_HUMAN	-0.634628
PCNA_HUMAN	1.92967		P63_HUMAN	-9.87507
SP1_HUMAN	1.61246		CSPG2_HUMAN	-4.44
BCL2_HUMAN	3.49745		SNAI2_HUMAN	-3.76971
TSC2_HUMAN	0.85307		SPB5_HUMAN	-2.50339
FOXA1_HUMAN	4.50315		ASC_HUMAN	-8.65988
GDF15_HUMAN	2.69952		Z385A_HUMAN	-1.76185
EDN2_HUMAN	3.10815		1433S_HUMAN	-4.25544
DDB2_HUMAN	2.26436		PERP_HUMAN	-3.34831
XPO2_HUMAN	1.21701		DKK1_HUMAN	-3.13909
E2F1_HUMAN	5.13942		MDM2_HUMAN	-1.01875
ASPP2_HUMAN	0.965443		CD82_HUMAN	-4.86454
APAF_HUMAN	1.06935		R144B_HUMAN	-3.6513
IBP3_HUMAN	6.04069		BTG2_HUMAN	-4.20738
E2F2_HUMAN	6.42348		BKRB2_HUMAN	-2.06574
			TIGAR_HUMAN	-2.66362
			GA45A_HUMAN	-3.12129
			TGFA_HUMAN	-2.43847
			CAV1_HUMAN	-5.35133
			BAK_HUMAN	-2.04026
			IRF5_HUMAN	-3.13589
			T53I1_HUMAN	-6.09755

Table S7: list of over and under expressed transcripts members of the regulatory network "Direct effectors of p53"

pathway name	set size	candidates contained	p-value	q-value
Steroid biosynthesis	17	10 (58.8%)	3.71E-06	0.000775
Tight junction	132	33 (25.0%)	1.17E-05	0.00122
Endocytosis	202	44 (21.8%)	2.07E-05	0.00144
Adherens junction	73	20 (27.4%)	0.00016	0.00836
alpha-Linolenic acid metabolism	21	9 (42.9%)	0.000287	0.0102
Axon guidance	129	29 (22.5%)	0.000292	0.0102
Cell adhesion molecules (CAMs)	133	29 (21.8%)	0.000502	0.0143
Arachidonic acid metabolism	63	17 (27.0%)	0.00059	0.0143
Salmonella infection	86	21 (24.4%)	0.000618	0.0143
VEGF signaling pathway	76	19 (25.0%)	0.000811	0.0161
Amoebiasis	108	24 (22.6%)	0.000847	0.0161
Fc gamma R-mediated phagocytosis	97	22 (23.2%)	0.000999	0.0168
Pathogenic Escherichia coli infection	55	15 (27.3%)	0.00108	0.0168
Regulation of actin cytoskeleton	212	40 (18.9%)	0.00112	0.0168
Terpenoid backbone biosynthesis	21	8 (38.1%)	0.00158	0.0221
Notch signaling pathway	47	13 (27.7%)	0.00199	0.026
Bladder cancer	42	12 (28.6%)	0.00217	0.0267
Phosphatidylinositol signaling system	80	18 (22.5%)	0.00387	0.045
Linoleic acid metabolism	29	9 (31.0%)	0.00416	0.0458
Focal adhesion	200	36 (18.0%)	0.0044	0.0459
Bacterial invasion of epithelial cells	70	16 (22.9%)	0.00534	0.0531
Glioma	65	15 (23.1%)	0.00626	0.0586
Leukocyte transendothelial migration	116	23 (19.8%)	0.00644	0.0586
Biosynthesis of unsaturated fatty acids	21	7 (33.3%)	0.00728	0.0634
Fc epsilon RI signaling pathway	81	17 (21.5%)	0.00787	0.0658

Table S8 Enriched KEGG pathway-based sets of under-expressed transcripts. Set size refers to the number of entities that have a Uniprot ID as stated in the corresponding KEGG pathway-based set at the ConsensusPathDB site. The number of candidates contained refers to amount of proteins which are part of the extended network and appear as part of the pathway. P-values are calculated according to a hypergeometric test; q-values represent p-values corrected for multiple testing using the false discovery rate method.

Gene Ontology term	Set size	Candidates contained	P-value	Q-value
GO:0043933 macromolecular complex subunit organization	1176	68 (5.8%)	1.74E-21	3.45E-19
GO:0071842 cellular component organization at cellular level	3203	116 (3.6%)	3.25E-20	3.24E-18
GO:0022607 cellular component assembly	1520	72 (4.7%)	7.10E-18	4.71E-16
GO:0007018 microtubule-based movement	144	18 (12.5%)	1.27E-11	6.31E-10
GO:0019058 viral infectious cycle	235	21 (9.0%)	1.43E-10	5.71E-09
GO:0046907 intracellular transport	1101	45 (4.1%)	3.43E-09	1.14E-07
GO:0072594 establishment of protein localization to organelle	206	18 (8.8%)	4.35E-09	1.24E-07
GO:0035966 response to topologically incorrect protein	139	15 (10.8%)	5.31E-09	1.29E-07
GO:0045104 intermediate filament cytoskeleton organization	27	8 (29.6%)	5.86E-09	1.29E-07
GO:0022411 cellular component disassembly	291	21 (7.2%)	7.09E-09	1.41E-07
GO:0019080 viral genome expression	155	15 (9.7%)	2.17E-08	3.59E-07
GO:0019083 viral transcription	155	15 (9.7%)	2.17E-08	3.59E-07
GO:0015031 protein transport	1216	44 (3.6%)	1.82E-07	2.79E-06
GO:0044260 cellular macromolecule metabolic process	6676	144 (2.2%)	1.35E-06	1.92E-05
GO:0008104 protein localization	1523	48 (3.2%)	2.59E-06	3.44E-05
GO:0070271 protein complex biogenesis	755	30 (4.0%)	3.24E-06	4.03E-05
GO:0019047 provirus integration	8	4 (50.0%)	4.01E-06	4.43E-05
GO:0030069 lysogeny	8	4 (50.0%)	4.01E-06	4.43E-05
GO:0070727 cellular macromolecule localization	895	33 (3.7%)	4.92E-06	5.15E-05
GO:0071843 cellular component biogenesis at cellular level	245	15 (6.1%)	8.33E-06	8.29E-05
GO:0019538 protein metabolic process	3827	88 (2.3%)	6.38E-05	0.000605
GO:0044248 cellular catabolic process	1683	47 (2.8%)	7.48E-05	0.000677
GO:0016052 carbohydrate catabolic process	170	11 (6.5%)	8.20E-05	0.000709
GO:0044282 small molecule catabolic process	319	15 (4.7%)	0.000172	0.00142
GO:0048731 system development	3281	75 (2.3%)	0.000347	0.00276
GO:0006139 nucleobase-containing compound metabolic process	5195	108 (2.1%)	0.000437	0.00335
GO:0034641 cellular nitrogen compound metabolic process	5621	115 (2.0%)	0.000478	0.00353
GO:0019059 initiation of viral infection	25	4 (16.0%)	0.000586	0.00403
GO:0007596 blood coagulation	518	19 (3.7%)	0.000588	0.00403
GO:0007599 hemostasis	522	19 (3.6%)	0.000645	0.00428
GO:0009057 macromolecule catabolic process	844	26 (3.1%)	0.000859	0.00552
GO:0016192 vesicle-mediated transport	945	28 (3.0%)	0.00101	0.00625
GO:0000086 G2/M transition of mitotic cell cycle	145	8 (5.5%)	0.00212	0.0128
GO:0022614 membrane to membrane docking	5	2 (40.0%)	0.00239	0.0138
GO:0030154 cell differentiation	2562	58 (2.3%)	0.00243	0.0138
GO:0030168 platelet activation	222	10 (4.5%)	0.0028	0.0155

**Swisprot ID**	**Rep_1**	**Mass_ 1**	**Score_ 1**	**Matches_ 1**	**Rep_2**	**Mass_2**	**Score_ 2**	**Matches_ 2**	**Rep_3**	**Mass_ 3**	**Score_3**	**Matches_ 3**
1433B									1433B	28179	78	5(2)
1433E					1433E	29326	47	2(1)	1433E	29326	128	3(3)
1433F					1433F	28372	49	2(2)				
1433G					1433G	28456	47	3(1)	1433G	28456	78	4(2)
1433S					1433S	27871	49	2(2)				
1433Z					1433Z	27899	136	5(4)	1433Z	27899	140	7(4)
4F2									4F2	68180	52	1(1)
ACDSB	ACDSB	47797	34	1(1)								
ACOXL					ACOXL	62383	37	1(1)	ACOXL	62383	44	2(1)
ACTA	ACTA	42381	37	1(1)								
ACTB					ACTB	42052	76	3(2)	ACTB	42052	401	13(11)
ACTBL					ACTBL	42318	63	4(1)	ACTBL	42318	161	4(4)
ACTBM					ACTBM	42331	60	2(1)				
ACTC									ACTC	42334	223	9(6)
ACTN1									ACTN1	103563	50	1(1)
AHNK					AHNK	629213	83	3(2)				
AHNK2									AHNK2	617383	47	1(1)
AINX	AINX	55391	42	1(1)								
AKTS1									AKTS1	27595	35	1(1)
ALBU									ALBU	71317	73	3(1)
ALDOA					ALDOA	39851	77	2(1)	ALDOA	39851	187	7(5)
ANO8												
ANXA2					ANXA2	38808	54	1(1)	ANXA2	38808	108	4(4)
ANXA5	ANXA5	35971	59	2(1)								
AP3D1					AP3D1	131159	36	1(1)				
ASAH1					ASAH1	45087	33	1(1)				
ASAP2					ASAP2	112835	47	1(1)				
ASSY									ASSY	46786	44	1(1)
ATR					ATR	304764	38	2(1)				

HBB					HBB	16102	46	2(1)				
HDGF					HDGF	26886	68	4(3)	HDGF	26886	75	4(2)
HIP1R									HIP1R	119999	36	1(1)
HMGA1									HMGA 1	11669	64	1(1)
HMMR					HMMR	84448	34	1(1)				
HNRCL	HNRCL	32180	75	1(1)								
HNRPC									HNRPC	33707	106	3(3)
HNRPD									HNRPD	38581	38	2(1)
HNRPK					HNRPK	51230	53	2(1)	HNRPK	51230	133	4(4)
HNRPU					HNRPU	91269	116	6(5)	HNRPU	91269	253	13(6)
HPPD									HPPD	45077	34	1(1)
HS71L	HS71L	70730	72	3(1)								
HS902					HS902	39454	76	6(2)				
HS90A					HS90A	85006	254	8(7)	HS90A	85006	555	22(19)
HS90B					HS90B	83554	328	18(11)	HS90B	83554	942	36(27)
HSP71					HSP71	70294	92	4(2)	HSP71	70294	240	12(9)
HSP76					HSP76	71440	40	1(1)	HSP76	71440	195	6(6)
HSP7C					HSP7C	71082	34	1(1)	HSP7C	71082	330	13(10)
HSPB1					HSPB1	22826	59	2(2)	HSPB1	22826	79	3(2)
HTSF1					HTSF1	86371	39	1(1)				
HUWE1									HUWE 1	485523	35	2(1)
IF1AX					IF1AX	16564	41	2(1)				
IF1AY									IF1AY	16546	78	2(1)
IF4A1					IF4A1	46353	67	3(1)	IF4A1	46353	73	4(2)
IF4B									IF4B	69167	96	3(1)
ILF2									ILF2	43263	80	2(2)
JKIP1									JKIP1	73506	37	1(1)
K1C10					K1C10	59020	111	5(3)	K1C10	59020	399	16(12)
K1C12					K1C12	53592	39	2(1)				

K1C13									K1C13	49900	64	2(1)
K1C14					K1C14	51872	88	3(2)	K1C14	51872	177	6(6)
K1C15	K1C15	49409	53	3(2)								
K1C16									K1C16	51578	187	7(7)
K1C17					K1C17	48361	35	3(1)	K1C17	48361	103	5(3)
K1C18	K1C18	48029	80	3(2)								
K1C19	K1C19	44079	89	4(3)								
K1C25					K1C25	49858	59	2(1)				
K1C27					K1C27	50419	72	2(1)				
K1C28	K1C28	51163	42	3(1)								
K1C9					K1C9	62255	203	7(4)	K1C9	62255	60	2(1)
K1H1					K1H1	48633	45	2(1)				
K22E					K22E	65678	87	5(2)	K22E	65678	104	7(3)
K22O					K22O	66370	44	4(1)	K22O	66370	94	4(4)
K2C1					K2C1	66170	350	13(11)	K2C1	66170	282	11(9)
K2C1B					K2C1B	62149	40	2(1)				
K2C4					K2C4	57649	93	3(2)	K2C4	57649	65	2(1)
K2C5					K2C5	62568	33	1(1)	K2C5	62568	175	10(8)
K2C6A					K2C6A	60293	44	3(1)				
K2C6B					K2C6B	60315	67	3(2)	K2C6B	60315	88	4(2)
K2C6C									K2C6C	60273	253	15(11)
K2C7					K2C7	51411	66	2(1)				
K2C73					K2C73	59457	36	2(1)				
K2C75					K2C75	59809	57	3(1)				
K2C79					K2C79	58085	60	2(1)	K2C79	58085	118	6(5)
K2C8					K2C8	53671	86	3(1)	K2C8	53671	63	4(2)
K2C80	K2C80	51007	78	2(1)								
KCNG3					KCNG3	50359	34	1(1)				
KI20B					KI20B	211868	36	1(1)				
KIF1A					KIF1A	192541	36	2(1)				
KPYM									KPYM	58470	61	1(1)

BCL7B	BCL7B	22195	37	1(1)								
BFSP1					BFSP1	74784	44	1(1)				
BLK									BLK	58126	51	1(1)
BZW1									BZW1	48184	44	2(1)
BZW2					BZW2	48360	47	1(1)	BZW2	48360	54	2(2)
C1QBP	C1QBP	31742	92	2(2)								
CALR	CALR								CALR	48283	35	1(1)
CALU	CALU	37198	36	3(1)								
CALX									CALX	67982	49	1(1)
CAPS2					CAPS2	148895	34	1(1)				
CC110	CC110	96726	49	2(1)								
CDK1									CDK1	34131	51	1(1)
CH60												
CH60					CH60	61187	103	3(3)	CH60	61187	173	9(6)
CHM2A									CHM2A	25088	37	1(1)
CHMP5									CHMP5	24612	35	1(1)
CHRC1									CHRC1	14758	36	1(1)
CLIC1									CLIC1	27248	41	1(1)
CNTLN									CNTLN	162131	37	1(1)
CNTP4					CNTP4	147178	37	1(1)	CNTP4	147178	37	1(1)
CO039	CO039	112086	35	2(1)								
CO6A1	CO6A1	108529	39	1(1)								
CTNA1									CTNA1	100693	38	1(1)
DDX21									DDX21	87804	88	4(3)
DIRA2	DIRA2	22813	52	1(1)								
DNJA2	DNJA2	46344	47	1(1)								
DPF3	DPF3	44254	37	1(1)								
DX39A	DX39A	49611	54	4(2)								
DYH2	DYH2	510796	39	1(1)								
EBP2									EBP2	34887	36	1(1)

EEPD1	EEPD1	62820	43	1(1)								
EF1A1					EF1A1	50451	59	1(1)	EF1A1	50451	138	7(4)
EF1B					EF1B	24919	106	3(3)	EF1B	24919	139	2(2)
EF1D					EF1D	31217	39	1(1)	EF1D	31217	283	6(6)
EFTU					EFTU	49852	34	1(1)	EFTU	49852	44	4(1)
EIF3B					EIF3B	92823	59	1(1)	EIF3B	92823	224	10(8)
EIF3J					EIF3J	29159	60	1(1)	EIF3J	29159	45	2(1)
ENOA	ENOA	47481	41	1(1)	ENOA	47481	66	2(1)	ENOA	47481	135	7(6)
ENPL	ENPL	92696	71	7(2)								
EZRI									EZRI	69484	50	4(2)
F10A1					F10A1	41477	65	3(2)	F10A1	41477	200	8(4)
F16A1									F16A1	117744	36	2(1)
G3BP1					G3BP1	52189	49	4(3)	G3BP1	52189	326	11(10)
G3BP2					G3BP2	54145	47	3(2)	G3BP2	54145	60	3(2)
G3P					G3P	36201	64	3(2)	G3P	36201	223	5(3)
G3PT	G3PT	44815	62	2(2)								
GBF1					GBF1	208367	45	2(1)				
GBRA5	GBRA5	52398	33	1(0)								
GFAP					GFAP	49907	56	2(1)				
GIT2	GIT2	85117	51	6(3)								
GLU2B					GLU2B	60357	56	1(1)	GLU2B	60357	71	3(1)
GOGA1	GOGA1	88244	42	1(1)								
GP128					GP128	89992	35	1(1)				
GRP75									GRP75	73920	46	1(1)
GRP78					GRP78	72402	142	7(4)	GRP78	72402	235	10(10)
H33									H33	15376	108	11(5)
H3C					H3C	15318	45	4(2)	H3C	15318	47	2(1)
H4	H4	11367	45	2(1)								
H90B2					H90B2	44492	86	4(3)	H90B2	44492	245	12(8)
H90B3									H90B3	68624	346	14(11)
H90B4					H90B4	58855	64	5(3)	H90B4	58855	45	3(1)

KRT35					KRT35	51640	45	3(1)				
KRT38	KRT38	52044	56	2(1)								
KT222	KT222	34158	68	2(1)								
LA									LA	46979	51	1(1)
LC7L2									LC7L2	46942	48	1(1)
LDH6A									LDH6A	36826	91	2(1)
LDHA									LDHA	36950	110	5(3)
LDHB									LDHB	36900	125	5(5)
LEG3									LEG3	26152	39	1(1)
LIMA1					LIMA1	85630	52	3(1)	LIMA1	85630	47	2(1)
LMNA					LMNA	74380	57	1(1)	LMNA	74380	215	7(6)
LMNB1									LMNB1	66653	39	3(2)
LMNB2									LMNB2	67762	39	2(2)
MAGD4	MAGD4	81555	43	1(1)								
MATR3					MATR3	95078	78	5(3)	MATR3	95078	38	4(1)
MCM2									MCM2	102516	40	1(1)
MCM3					MCM3	91551	46	1(1)	MCM3	91551	117	4(3)
MDHM	MDHM	35937	35	1(1)								
MIF									MIF	12639	54	1(1)
MMP3									MMP3	54228	36	2(1)
MOES									MOES	67892	50	5(2)
MYH15					MYH15	225904	43	1(1)				
MYO1A	MYO1A	119238	39	1(1)								
NACA					NACA	23370	214	4(4)	NACA	23370	320	6(6)
NACA2					NACA2	23209	104	1(1)	NACA2	23209	130	2(2)
NACAD									NACAD	161971	66	1(1)
NFM												
NOP2									NOP2	89589	42	1(1)
NPM					NPM	32726	65	6(3)	NPM	32726	597	18(12)
OVCH1					OVCH1	127095	36	1(1)				
PA2G4					PA2G4	44101	35	2(1)				

PARK7									PARK7	20050	36	2(1)
PCBP1									PCBP1	37987	32	1(0)
PCNP									PCNP	18913	37	2(1)
PDIA1					PDIA1	57480	649	27(20)	PDIA1	57480	884	31(28)
PDIA4					PDIA4	73229	37	1(1)	PDIA4	73229	452	22(16)
PDIA6	PDIA6	48490	62	2(1)								
PERI	PERI	53651	90	3(1)								
PGK1									PGK1	44985	78	4(2)
PGK2									PGK2	45166	79	1(1)
PHB					PHB	29843	39	2(1)				
PHLA1	PHLA1	45501	35	1(1)								
PKHG1	PKHG1	156541	30	1(1)								
PKHG5									PKHG5	118974	36	1(1)
PLCL2									PLCL2	127268	36	1(1)
POTEE					POTEE	122882	90	2(1)	POTEE	122882	256	6(5)
POTEI									POTEI	122858	126	5(3)
PPIA					PPIA	18229	50	5(2)	PPIA	18229	210	8(6)
PPIB					PPIB	23785	80	2(1)	PPIB	23785	271	5(2)
PRDM8									PRDM8	72588	34	1(1)
PRDX1					PRDX1	22324	117	5(4)	PRDX1	22324	137	9(6)
PRDX3					PRDX3	28017	58	1(1)	PRDX3	28017	44	3(1)
PRDX4									PRDX4	30749	39	2(1)
PRDX6					PRDX6	25133	42	1(1)	PRDX6	25133	99	4(3)
PROF1					PROF1	15216	75	3(2)	PROF1	15216	163	5(5)
PSA3					PSA3	28643	78	2(1)	PSA3	28643	172	6(6)
PSIP1									PSIP1	60181	70	3(1)
PVRL4									PVRL4	55933	34	1(1)
RAB1A									RAB1A	22891	52	1(1)
RAB28					RAB28	25054	36	1(1)	RAB28	25054	39	1(1)
RAB35									RAB35	23296	52	1(1)
RALY					RALY	32501	66	4(1)	RALY	32501	111	5(5)

RALYL					RALYL	32425	66	1(1)	RALYL	32425	71	1(1)
RAN					RAN	24579	87	4(2)	RAN	24579	39	1(1)
RBBP4	RBBP4	47911	44	4(1)								
RBBP5	RBBP5	59800	33	1(0)								
RBBP7	RBBP7	48132	88	3(2)								
RL11									RL11	20468	86	2(1)
RL22					RL22	14835	181	4(4)	RL22	14835	157	2(2)
RL7									RL7	29264	52	2(1)
RL8					RL8	28235	35	1(1)				
RLA0					RLA0	34423	111	4(3)	RLA0	34423	258	6(6)
RLA0L					RLA0L	34514	48	1(1)				
RLA1					RLA1	11621	41	1(1)				
RLA2					RLA2	11658	63	2(1)	RLA2	11658	206	5(5)
ROA3									ROA3	39799	34	1(1)
RS14					RS14	16434	35	1(1)				
RS15A									RS15A	14944	36	1(1)
RS16					RS16	16549	49	1(1)				
RS28									RS28	7893	35	1(1)
RS3A									RS3A	30154	43	4(1)
RS4X					RS4X	29807	61	1(1)				
RS8					RS8	24475	85	2(2)				
RU17					RU17	51583	41	1(1)				
SAE2									SAE2	71749	56	3(2)
SAFB1									SAFB1	103036	40	2(1)
SARNP									SARNP	23713	52	3(2)
sep-02					sep-02	41689	59	1(1)	sep-02	41689	149	2(2)
SF3A1					SF3A1	88888	75	7(4)	SF3A1	88888	238	15(12)
SF3B2					SF3B2	100279	139	3(2)	SF3B2	100279	36	1(1)
SNED1					SNED1	158206	33	1(1)	SNED1	158206	36	1(1)
SPTN4	SPTN4	290005	42	1(1)								
SRSF1					SRSF1	27842	57	1(1)	SRSF1	27842	109	4(2)

SRSF3					SRSF3	19546	91	3(3)	SRSF3	19546	53	3(1)
ST134									ST134	27561	53	4(1)
TAGL2									TAGL2	22548	56	1(1)
TBA1A					TBA1A	50788	59	1(1)	TBA1A	50788	43	1(1)
TBA1B					TBA1B	50804	368	13(11)	TBA1B	50804	586	22(18)
TBA1C	TBA1C	50548	363	17(10)								
TBA3E					TBA3E	50568	105	6(4)				
TBA4A					TBA4A	50634	218	9(7)				
TBA4B									TBA4B	27819	202	4(4)
TBA8					TBA8	50746	150	7(5)	TBA8	50746	262	10(7)
TBB1					TBB1	50865	148	5(4)	TBB1	50865	124	7(4)
TBB2A					TBB2A	50274	57	1(1)	TBB2A	50274	599	21(14)
TBB2C	TBB2C	50255	98	5(3)								
TBB3					TBB3	50856	90	2(2)	TBB3	50856	105	4(3)
TBB4B					TBB4B	50255	566	19(14)	TBB4B	50255	722	25(17)
TBB5					TBB5	50095	777	20(24)	TBB5	50095	936	30(21)
TBB8	TBB8	50257	103	5(2)								
TCP4									TCP4	14386	139	4(1)
TENS1					TENS1	186499	38	1(1)				
TPIS					TPIS	31057	75	2(1)	TPIS	31057	68	4(2)
TRAP1					TRAP1	80345	96	2(1)	TRAP1	80345	54	1(1)
TSNA1	TSNA1	56655	40	1(1)								
UN13C					UN13C	252693	39	2(1)				
UTS2									UTS2	14515	39	1(1)
VIME					VIME	53676	100	1(1)	VIME	53676	344	10(8)
WNT7A					WNT7A	40405	34	1(1)				
XRCC6									XRCC6	70084	53	4(1)
ZN275	ZN275	49780	34	1(1)								
ZN524					ZN524	29262	44	1(1)				

Table S9 Phosphoproteins relationship according to which replicates were identified

GO:0000226	microtubule cytoskeleton organization	272	11 (4.0%)	0.00402	0.0216
GO:0012501	programmed cell death	1551	38 (2.5%)	0.0042	0.022
GO:0051235	maintenance of location	173	8 (4.6%)	0.00619	0.0316
GO:0048519	negative regulation of biological process	2999	63 (2.1%)	0.00862	0.0429
GO:0009611	response to wounding	1105	28 (2.5%)	0.00888	0.0431

Table S10 Enriched gene ontology level 3 categories of biological processes of identified phosphoproteins. Set size refers to the number of entities that have a UniProt ID as stated in the corresponding GO category at the ConsensusPathDB site. The number of candidates contained refers to amount of proteins identified in this study that appear as part of the GO category. P-values are calculated according to a hypergeometric test; q-values represent p-values corrected for multiple testing using the false discovery rate method.

Pathway name	Set size	Candidates contained	P-value	Q-value
Signaling events mediated by HDAC Class III	40	17 (43.6%)	5.03E-19	2.57E-17
p38 signaling mediated by MAPKAP kinases	22	7 (33.3%)	1.45E-07	3.38E-06
LKB1 signaling events	45	9 (20.9%)	1.99E-07	3.38E-06
Validated targets of C-MYC transcriptional activation	87	11 (13.6%)	8.79E-07	1.12E-05
Class I PI3K signaling events mediated by Akt	36	7 (20.0%)	6.55E-06	5.64E-05
mTOR signaling pathway	65	9 (14.1%)	6.64E-06	5.64E-05
Insulin-mediated glucose transport	31	6 (20.0%)	3.07E-05	0.000224
FoxO family signaling	50	7 (14.3%)	6.52E-05	0.000416
Trk receptor signaling mediated by PI3K and PLC-gamma	37	6 (16.7%)	9.07E-05	0.000514
Role of Calcineurin-dependent NFAT signaling in lymphocytes	57	7 (13.0%)	0.000123	0.000576
Signaling events mediated by HDAC Class II	39	6 (15.8%)	0.000124	0.000576
a6b1 and a6b4 Integrin signaling	46	6 (13.3%)	0.000324	0.00138
Regulation of nuclear beta catenin signaling and target gene transcription	81	7 (8.8%)	0.0014	0.00548
Aurora B signaling	43	5 (11.9%)	0.00174	0.00635
Integrin-linked kinase signaling	46	5 (11.1%)	0.00238	0.00802
HIF-1-alpha transcription factor network	66	6 (9.1%)	0.00252	0.00802
Regulation of Telomerase	73	6 (8.8%)	0.00293	0.00878
Caspase cascade in apoptosis	53	5 (9.4%)	0.00489	0.0138
PDGFR-beta signaling pathway	130	8 (6.3%)	0.00518	0.0139
Aurora C signaling	7	2 (33.3%)	0.0062	0.0158
ATR signaling pathway	37	4 (10.8%)	0.0072	0.0175

Table S11 Enriched PID pathway-based sets of identified phosphoproteins. Set size refers to the number of entities that have a Uniprot ID as stated in the corresponding PID pathway-based set at the ConsensusPathDB site. The number of candidates contained refers to amount of proteins which are part of the extended network and appear as part of the pathway. P-values are calculated according to a hypergeometric test; q-values represent p-values corrected for multiple testing using the false discovery rate method.

**Pathway name**	**Set size**	**Candidates contained**	**P-value**	**Q-value**
Pathogenic Escherichia coli infection	55	16 (29.1%)	1.16E-14	7.33E-13
Systemic lupus erythematosus	138	19 (14.0%)	5.10E-11	1.61E-09
Ribosome	91	14 (15.6%)	4.66E-09	9.79E-08
Gap junction	89	13 (14.6%)	3.68E-08	5.80E-07
Spliceosome	127	15 (11.8%)	6.07E-08	7.65E-07
Glycolysis / Gluconeogenesis	65	11 (16.9%)	8.96E-08	9.41E-07
Alcoholism	179	17 (9.5%)	2.00E-07	1.80E-06
Protein processing in endoplasmic reticulum	166	16 (9.6%)	3.79E-07	2.99E-06
Phagosome	154	15 (9.9%)	6.56E-07	4.59E-06
Antigen processing and presentation	76	10 (13.2%)	3.85E-06	2.43E-05
Legionellosis	55	8 (14.5%)	1.73E-05	9.92E-05
Epstein-Barr virus infection	203	14 (7.0%)	8.43E-05	0.000443
Cell cycle	124	10 (8.1%)	0.000275	0.00133
Pyruvate metabolism	41	5 (12.2%)	0.00158	0.00712
Propanoate metabolism	32	4 (12.5%)	0.0043	0.0181
Phenylalanine metabolism	18	3 (16.7%)	0.00592	0.0233

Table S12 Enriched KEGG pathway-based sets of identified phosphoproteins. Set size refers to the number of entities that have a Uniprot ID as stated in the corresponding KEGG pathway-based set at the ConsensusPathDB site. The number of candidates contained refers to amount of proteins which are part of the extended network and appear as part of the pathway. P-values are calculated according to a hypergeometric test; q-values represent p-values corrected for multiple testing using the false discovery rate method.
